# Rescue of lysosomal acid lipase deficiency in mice by rAAV8 liver gene transfer

**DOI:** 10.1038/s43856-025-00816-8

**Published:** 2025-04-11

**Authors:** Marine Laurent, Rim Harb, Christine Jenny, Julie Oustelandt, Simon Jimenez, Jeremie Cosette, Francesca Landini, Aristide Ferrante, Guillaume Corre, Nemanja Vujic, Claudia Piccoli, Anais Brassier, Laetitia Van Wittenberghe, Giuseppe Ronzitti, Dagmar Kratky, Consiglia Pacelli, Mario Amendola

**Affiliations:** 1 Genethon, 91000 Evry, France; 2 Paris-Saclay University, Univ Evry, Inserm, Genethon, Integrare research unit UMR_S951, 91000 Evry, France; 3 Department of Clinical and Experimental Medicine, University of Foggia, Foggia, Italy; 4 Gottfried Schatz Research Center, Molecular Biology and Biochemistry, Medicine University of Graz, Graz, Austria; 5 BioTechMed-Graz, Graz, Austria; 6 Necker-Enfants-Malades University Hospital, Paris, France

**Keywords:** Medical research, Metabolic disorders

## Abstract

**Background:**

Lysosomal acid lipase deficiency (LAL-D) is an autosomal recessive disorder caused by mutations in the *LIPA* gene, which results in lipid accumulation leading to multi-organ failure. If left untreated, the severe form of LAL-D results in premature death within the first year of life due to failure to thrive and hepatic insufficiency. Weekly systemic injections of recombinant LAL protein, referred as enzyme replacement therapy, is the only available supportive treatment.

**Method:**

Here, we characterized a novel *Lipa*^*−/−*^ mouse model and developed a curative gene therapy treatment based on the in vivo administration of recombinant (r)AAV8 vector encoding the human *LIPA* transgene under the control of a hepatocyte-specific promoter.

**Results:**

Here we define the minimal rAAV8 dose required to rescue disease lethality and to correct cholesterol and triglyceride accumulation in multiple organs and blood. Finally, using liver transcriptomic and biochemical analysis, we show mitochondrial impairment in *Lipa*^*−/−*^ mice and its recovery by gene therapy.

**Conclusions:**

Overall, our in vivo gene therapy strategy achieves a stable long-term LAL expression sufficient to correct the disease phenotype in the *Lipa*^*−/−*^ mouse model and offers a new therapeutic option for LAL-D patients.

## Plain language summary

Lysosomal acid lipase (LAL) deficiency is a rare genetic disorder that causes toxic lipid build-up in liver and other organs and causes death during childhood if untreated. This study aimed to develop a treatment to cure this disorder by introducing a healthy copy of the LAL gene (gene that causes the disorder). We tested this in a mouse model that lacks this gene and has symptoms similar to the human disorder. We found that this treatment could effectively prevent disease symptoms and reverse damage in affected tissues. This promising gene therapy could offer a potential long-term cure for LAL deficiency, providing hope for patients with this life-threatening condition in the future.

## Abstract (web summary)

Laurent et al. develop a liver-targeted gene therapy using recombinant adeno-associated viral vector (rAAV) to effectively cure Lysosomal acid lipase deficiency. This occurs via correction of lipid accumulation and normalized gene expression patterns and mitochondrial function in *Lipa*^−/−^ mice.

## Introduction

Lysosomal acid lipase deficiency (LAL-D) is a rare autosomal recessive lysosomal storage disorder caused by mutations in the *LIPA* gene. In healthy lysosomes, LAL is the sole enzyme responsible for the hydrolysis of cholesterol esters and triglycerides into free cholesterol and fatty acids respectively, necessary for energy production, membrane formation and biosynthesis of cholesterol-derived compounds. In LAL-D patients, cholesterol esters and triglycerides accumulate in tissues and organs, especially in liver, spleen, and small intestine, resulting in multi-organ failure. As a consequence, LAL-D patients suffer from hepatosplenomegaly, elevated circulating alanine (ALT) and aspartate (AST) transaminases due to liver damage and hyperlipidemia with elevated low-density (LDL) and reduced high-density lipoprotein (HDL). Unlike other lysosomal storage disorders, LAL-D does not obviously affect the central nervous system^1,2^. The severity of the disorder depends on the residual amount of functional LAL enzyme. Two forms of the disease have been described: infantile-onset, formerly known as Wolman disease (WD, OMIM 620151; prevalence ~0.325–1.11 cases per million births), with <2% of remaining functional LAL^3,4^; childhood/adult-onset LAL-D, formerly known as cholesteryl ester storage disease (CESD, OMIM 278000; prevalence 3.13–4.86 cases per million births), with 2–12% of remaining functional LAL. WD patients have difficulty to thrive, intestinal malabsorption, adrenal gland calcification and experience premature death in the first year after birth without treatment. In contrast, CESD may manifest in childhood or adulthood with a wide range of symptoms and severity, from asymptomatic to premature death if left untreated^4–6^.

Several therapeutic strategies have been attempted, including hematopoietic stem cells (HSC) and liver transplantation from healthy donors, but they were not effective in preventing disease progression and are limited by high procedure morbidity and HLA matched donor availability^5,7,8^. Nowadays, enzyme replacement therapy (ERT) with weekly intravenous administrations of recombinant LAL enzyme Sebelipase (Kanuma®, Alexion Pharmaceuticals, Inc.), is the standard care for LAL-D. This non-curative treatment improves patients’ lifespan and major clinical symptoms. However, it is associated with the development of antibodies against the recombinant enzyme in a fraction of treated patients, it remains very costly and its long-term efficacy is still under evaluation^9,10^.

In vivo gene therapy (GT) based on the delivery of a functional copy of the *LIPA* transgene represents an appealing curative treatment to continuously express therapeutic LAL enzyme. A proof-of-principle was previously obtained by intravenous injection of first-generation adenoviral vectors (AdV) encoding for human *LIPA* cDNA under the control of the viral CMV promoter into *Lipa*^*−/−*^ mice^11^. Transduced hepatocytes expressed and secreted human LAL into the circulation to cross-correct all affected tissues, but the effect was transient and a strong AdV immune response was observed.

To address these constraints, we assess a liver-based in vivo GT for LAL-D using recombinant single-stranded adeno associated viral vector (rAAV) 8 encoding for *LIPA* cDNA under the control of liver promoter. As animal model, we describe a new *Lipa*^*−/−*^ mouse characterised by reduced growth, body weight and lifespan (6-7 months) compared to their WT littermates. Upon rAAV8 injection, we establish the minimal vector dose required to achieve full correction of hepatosplenomegaly, haematological parameters, dyslipidaemia, and transcriptional deregulation, resulting in normal growth and lifespan of *Lipa*^*−/−*^ mice.

## Methods

### Study design

This study was designed to evaluate the therapeutic potential of liver-based in vivo gene therapy using rAAV8.hAAT.LIPA for LAL-D. We selected an *Lipa*^*−/−*^ mouse model that mimics the pathological lipids accumulation in the liver, spleen, and intestine, leading to reduced lifespan. To minimize the influence of sex on rAAV transduction^12^ and immune reactions^13^, male *Lipa*^*−/−*^ mice were chosen for the study. We initially evaluated the minimal doses of rAAV8 able to correct hematological compartment, hepatosplenomegaly, lipid accumulation and tissues architecture. Each group consisted of four animals, which were monitored for 12-weeks post-administration. After identifying the most suitable doses of rAAV8, the corrective effects were evaluated at three different time points: 4-, 12-, and 34-weeks post-administration. At each time point, a subset of mice was analyzed, including WT mice (3, 10 and 6 used respectively), *Lipa*^*−/−*^ mice (4, 5 and 4 used respectively), and rAAV8.hAAT.LIPA-treated *Lipa*^*−/−*^ mice (4, 6 and 6 used respectively). At the 34-weeks post-treatment, only one *Lipa*^*−/−*^ mouse remained alive, while the others were sacrificed upon reaching predefined endpoints set by the ethical committee CEEA-51. Random assignment of mice to experimental groups was conducted to minimize bias.

### Mice

Heterozygous Lipa^tm1a(EUCOMM)Hmgu/Biat^ mice were crossed with heterozygous mice expressing the Cre recombinase under the control of the CMV promotor to delete exon 4 of the mouse *LIPA* gene (Supplementary Fig. 1a). Homozygous mice were then backcrossed on the C57/BL6N background for >8 generations. Mice were maintained in a specific pathogen-free (SPF) environment with a regular light-dark (12 h/12 h) cycle and *ad libitum* access to food (standard chow) and water. This study was approved by the ethical committee CEEA-51 and conducted according to French and European legislation on animal experimentation (APAFIS #33620-2021101416506495). Genotyping was performed by PCR on DNA extracted from the tail using 50 mM NaOH and Tris-HCl pH8. KAPA2G Fast ReadyMix (Kapa Biosystem, Wilmington, MA, USA) was used for PCR with using 0.65 µmol of each primer (Table 1, purchased at Thermo Fisher Scientific Waltham, MA) and 1 µL of extracted DNA in a 12.5 µL reaction as per the manufacturer’s protocol. Genotyping bands were sequenced. Age and sex matched littermate controls were used for all experiments.

**Table 1:** List of primers

Gene	Name	Sequence (5’-3’)
LAL-WT	LAL F	GGCCTTTCACTTGCTTGGTT
	LAL-WT R	GGCTAAGCATGTACAGGAACA
LAL-KO	LAL-KO R	GGCCTGTCCCTCTCACCTTC
hAAT promoter	hAAT F	GGCGGGCGACTCAGATC
	hAAT P (FAM)	AGCCCCTGTTTGCTCCTCCGATAACTG
	hAAT R	GGGAGGCTGCTGGTGAATATT
Titin normalizer	Titin F	AAAACGAGCAGTGACGTGAGC
	Titin P (VIC)	TGCACGGAAGCGTCTCGTCTCAGTC
	Titin R	TTCAGTCATGCTGCTAGCGC

### Cloning and production of rAAV8

The cDNA of human *LIPA* gene (Gene ID:3988) with a C-terminal 3xHA tag^14^ was cloned in a standard rAAV2/8 vector backbone under the control of the hAAT promoter and optimized HBB2 intron^15^. All reagents and detailed sequence information are available upon request. Recombinant single-stranded rAAV2/8 was produced using an adenovirus-free triple transfection method of HEK293 and purified by single affinity chromatography (AVB Sepharose; GE Healthcare, Chicago, IL). The final product was formulated in sterile phosphate buffered saline containing 0.001% of Pluronic (F68; Sigma Aldrich, Saint Louis, MO), and stored at −80 °C^16,17^.

### Administration of rAAV8

rAAV8.hAAT.LIPA vectors were administered via tail vein injection to 10- to 12-week-old *Lipa*^*−/−*^ mice using an injection volume of 100 µL. The animals’ weights were measured the day before administration, and viral solutions were prepared according to the animals’ weight. Mice were weighed weekly until sacrifice. Every two- weeks after injection, blood was drawn after isoflurane anaesthesia (IsoFlurin, Axience) from the retro-orbital veins using a heparin-coated capillary and collected into Eppendorf tube containing 3,8% citrate. At 4-, 12- and 34-weeks post injection, mice were sacrificed by cervical dislocation and their tissues (liver, spleen, jejunum, thymus) were divided in two parts. One part was snap-frozen in isopentane (−160 °C) that was previously chilled in liquid nitrogen for histological analysis. The second part was disposed in 2 mL bead tubes type D (Machery Nagel, Düren, Germany) and frozen in liquid nitrogen to assess rAAV8 biodistribution and to quantify lipid accumulation and LAL enzymatic activity.

### Blood and plasma analyses

The blood samples were analysed for standard haematological parameters (WBC counts and distribution, RBC count, haemoglobin, MCV and platelet counts, MPV) using an MS9.5 counter (Melet Schloessing, Cergy-Pontoise, France). After these analyses, blood was centrifuged for 10 min at 1400 rcf and 4 °C to recover plasma, which was stored at −80 °C until further analysis. HDL, AST, and ALT levels were quantified in the plasma using specific colorimetric assays. A plasma control (Liquid assayed Multiqual; Bio-rad) was employed as a reference to quantify HDL, AST, and ALT. For the analysis, 10 μl of plasma were loaded onto specific chips designed for HDL, AST, and ALT measurements (HDL-C-P III D and GPT/ALT-P III; Fujifilm Europe Gmbh; FUJI DRI-CHEM NX500 - Sysmex). The concentration of HDL was measured in mg/dL, while ALT and AST were measured in U/L, providing quantitative data for these markers.

### Lipids analysis

Total lipids were extracted from 40 mg of frozen liver, spleen and jejunum using the chloroform-free Lipid Extraction Kit (Abcam, Waltham, MA, USA) according to the manufacturer’s protocol. Triglycerides were measured using the Triglycerides FS kit and total cholesterol was measured using the Cholesterol FS kit (both from DiaSys, Holzheim, Germany) following the manufacturer’s guidelines. Lipid concentrations were determined using a standard curve of triglycerides or cholesterol standards. Absorbance values were measured in duplicate using the EnSpire Multimode Plate Reader (Perkin Elmer, Waltham, MA, USA).

### Histological analysis

Serial 8 μm cross-sections of snap-frozen organs were cut on a Leica CM3050 S cryostat (Leica Biosystems). To minimise sampling errors, 2 sections of each specimen were obtained and stained with haematoxylin-eosin saffron (HES), Oil red O (ORO) and Sirius red (SR) according to standard procedures. For m(urine)CD68 staining, tissue sections were surrounded with a hydrophobic barrier using a hydrophobic pen (SIGMA Z377821) and dried or 5 min at RT. After blocking unspecific antigen using PBS (Gibco) and 10% of goat serum (Agilent X 0907) for 30 minutes at room temperature, slides were stained with rat anti-murine CD68 (dilution 1/500, Table 2) for 1 h at 4 °C. Then, slides with wash with sterile PBS (Gibco) 3 times and a secondary antibody Goat anti-Rat (Dilution 1/600, Table 2) was applied for 1 h at RT. After 3 washing steps in PBS, slides were mount using Fluoromount G + Dapi (Southern Biotech 0100-20). To minimise sampling errors, 2 sections of each specimen were obtained and stained.

**Table 2:** List of antibodies

Target	Host	Reference	Supplier
Anti-lysosomal acid lipase antibody	Mouse	ab154356	Abcam
Anti- α-actinin antibody	Mouse	sc-17829	Santa Cruz Biotechnology
Anti-mouse IgG (H + L) 800 CW	Goat	P/N: 926-32210	Li-Cor Biosciences
Anti-mouse IgG (H + L) 680 LT	Donkey	P/N: 926-68022	Li-Cor Biosciences
Rat anti-mouse CD68	Rat	MCA1957GA	Biorad
Goat anti-Rat IgG (H + L) Cross-Adsorbed Secondary Antibody, Alexa Fluor™ 594	Goat	A-11007	Invitrogen

Images of ORO, HES, SR and CD68 slides were digitised using an Axioscan Z1 slide scanner (Zeiss, Germany) with a Zeiss Plan-Apochromat 10X/0.45 M27 dry objective (ZEISS, Germany). Tile scan images were reconstructed with the ZEN software (ZEISS, Germany).

### ORO and SR quantification

ORO and SR images were processed using the pixel classification of QuPath 0.4.3^18^. For each staining and for each tissue, we created 2 classifiers, each time using several images for ground truth annotations. The first pixel classifier identifies pixels belonging to the tissue slice, excluding veins, folds, dust, bubbles, or other artifacts encountered, and draws an annotation of the analysable tissue. The second classifier was trained to identify fibrosis (for SR staining) or lipids (for ORO staining), based on manual ground truth annotations. This second classifier was then applied to the annotation created by the first classifier. The staining ratio for each tissue slice was obtained by dividing the total area of staining by the total surface area of the analysable tissue.

### mCD68 quantification

mCD68 images were processed using the pixel classification of QuPath 0.4.3^18^. For each staining and for each tissue, we created 2 classifiers, each time using several images for ground truth annotations. The first pixel classifier identifies pixels belonging to the tissue slice, excluding veins, folds, dust, bubbles, or other artifacts encountered, and draws an annotation of the analysable tissue. The second classifier was trained to identify mCD68 positive cells, based on manual ground truth annotations. This second classifier was then applied to the annotation created by the first classifier. The staining ratio for each tissue slice was obtained by dividing the total area of mCD68 staining by the total surface area of the analysable tissue.

### LAL enzyme activity assay

Frozen liver, spleen and jejunum were homogenized in cold sterile PBS (Gibco) using FastPrep (MP Biomedical, Ohio, USA) followed by centrifugation for 5 min at 1000xg and 4 °C. The collected supernatant was frozen at −80 °C prior to analysis. After sample processing, the frozen supernatant was centrifuged 20 min at 10,000×g to eliminate cell debris and the resulting supernatant was recovered. The protein content in the lysates was quantified using the BCA Protein Assay (Thermo Fisher Scientific). To determine LAL activity in liver and spleen, 1 µg of protein was used, whereas 10 µg of protein were used for the jejunum. Plasma was diluted 1/20 in sterile PBS prior to LAL activity measurement. Enzymatic activity was detected as previously described^14^. Briefly, samples were incubated for 10 min at 37 °C with 42 μM Lalistat-2 (Sigma-Aldrich, St. Louis, MI, USA) or water and then transferred to an Optiplate 96 F plate (PerkinElmer). Fluorometric reactions were initiated with 75 μL of substrate buffer (340 μM 4-MUP, 0.9% Triton X-100 and 220 μM cardiolipin in 135 mM acetate buffer pH 4.0). After 10 min, fluorescence was recorded (35 cycles, 30 sec intervals, 37 °C) using the SPARK TECAN Reader (Tecan, Austria). Kinetic parameters (average rate) were calculated using Magellan Software. LAL activity was quantified using Eq. (1).

1 $$\frac{{rAAV\; treated}\;{{{\rm{or}}}}\; {{{\rm{Lipa}}}}-/-{mice}\left({without\; lalistat}-{with\; lalistat}\right)}{{WT\; mice}\left({without\; lalistat}-{with\; lalistat}\right)}$$

### Western blot

Protein was extracted from frozen liver in sterile PBS using Fastprep crushing (MP Biomedical, Ohio, USA), followed by centrifugation for 10 min at 14,000×g and 4 °C to collect the supernatant. The protein content was quantified using BCA Protein Assay (Thermo Fisher Scientific) and 30 µg of protein were loaded as previously described^14^. α-actinin was used as loading control and antibodies are referred in Table 2. The blots were imaged at 169 μm with the Odyssey imager and analysed with ImageStudio Lite software (Li-Cor Biosciences, Lincoln, NE, USA). After image background subtraction (average method, top/bottom), the band intensities were quantified and normalised with α-actinin signal.

### Vector genomes

The genomic DNA was extracted from frozen organs using the DNA extraction kit (NucleoMag Pathogen; Macherey Nagel) and the KingFisher™ Flex Purification System with 96 Deep-Well Head (Thermo Fisher Scientific) following the manufacturer’s instructions. The number of vector copies per diploid genome was determined using Taqman qPCR with specific primers to amplify the hAAT promoter sequence with 100 ng of extracted DNA. Titin was used as normalizer to determine the number of genome copies. Data were expressed as vector genome copies per diploid genome.

### RNA seq

Azenta Life Science (Leipzig, Germany) received 40 mg of frozen liver for RNA sequencing. The paired-end reads (2 x 150bp) were aligned to the reference transcriptome (GRCm39/ensemble cDNA release 110) using SALMON v1.9.0^19^. Default parameters were used in addition to arguments -l A and –validate Mappings. The quantification files were processed using R (4.3.0) and the tximport package (1.28.0) to perform pairwise group differential expression analysis using DESeq2 (1.40.1) on genes with >100 reads across all samples (lfcThreshold = 0, pAdjustMethod = “fdr”, independentFiltering = T)^20^. Pathway enrichment analysis and GSEA were performed using the reactomePA (1.44.0) and clusterProfiler (4.8.1)^21^ packages for reactome^22^ and GO terms respectively. Genes were considered dysregulated if the absolute Log fold change was >1 and the FDR-adjusted *P* value < 0.05. The RNA-seq data, both raw and processed, are available on the GEO data set: GSE252742.

### Metabolic fluxes analysis in mouse liver

Mitochondria were isolated from frozen liver of mouse at 12- and 34-weeks post injection, as previously described^23^. Briefly, frozen liver tissue was minced and homogenized in MAS buffer containing 70 mM sucrose, 220 mM mannitol, 5 mM KH_2_PO_4_, 5 mM MgCl_2_, 1 mM EGTA and 2 mM HEPES pH 7.4. The homogenate was centrifuged at 1,000 × g for 5 min. The resulting supernatant was centrifuged at 10,000 × g for 10 min and the pellet was then resuspended in the same buffer. All centrifugation steps were carried out at 0–4 °C. Mitochondrial protein concentrations were determined using the Bradford method with bovine serum albumin as standard. The respiratory activity of freshly prepared mitochondria was measured with the XF96 extracellular flux analyser (Seahorse Bioscence, Agilent). Mitochondria (100 µg) were loaded into a Seahorse XF96 microplate in 20 μl of MAS. The loaded plate was centrifuged at 2000 g for 5 min at 4 °C (no brake) and an additional 130 μl of MAS containing cytochrome c (10 μg/ml, final concentration). The substrate was added as follows: NADH (2 mM), or succinate + rotenone (5 mM + 2 μM) was injected at port A; rotenone (2 µM) or rotenone + antimycin A (2 μM + 4 μM) at port B; TMPD + ascorbic acid (0.5 mM + 1 mM) at port C; and azide (50 mM) at port D. These conditions allowed for the determination of the respiratory capacity of mitochondria through Complex I, Complex II, and Complex IV.

### Statistics and reproducibility

The sample size (*n*) for each experiment is specified in the corresponding figure and in Supplementary Data 1. Each mouse represents a biological replicate, and all biochemical assays were conducted with two technical replicates. Statistical analyses were performed using GraphPad Prism version 9.00 for Windows (GraphPad Software, La Jolla, CA, USA, “www.graphpad.com”). One-way or two-way analysis of variance (ANOVA) with Tukey’s test or Bonferroni post-hoc analysis or Krustal-Wallis test were performed as indicated (alpha = 0.05). Values are expressed as mean ± standard deviation (SD). All unmarked comparisons reflect non-significant (*p* > 0.05) differences.

## Results

### rAAV8 injection improves body weight, haematological and plasma parameters at low dose

**Fig. 1:**
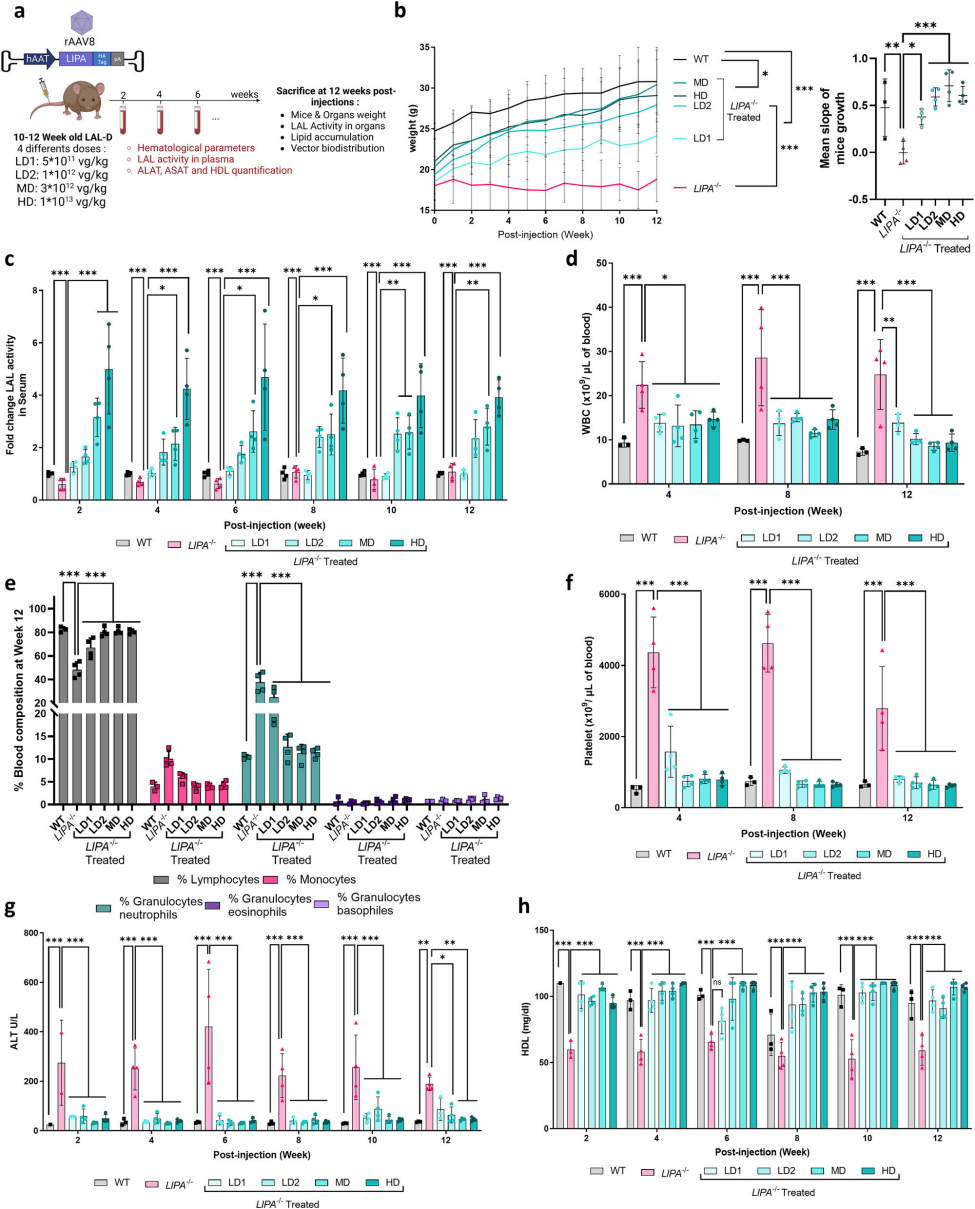
Evaluation of different rAAV8 doses in *Lipa*^−/−^ mice. **a** Experimental outline created by BioRender.com. 10–12-week-old mice were injected with the indicated rAAV8 doses and disease progression was monitored for 12-weeks post-injection. LD low dose, MD middle dose, HD high dose, vg vector genome. **b** Left panel: Mice weight (gr: grams) was recorded every week. Right panel: Linear slopes of mice weight. **c** Fold change of LAL activity over WT mice measured in plasma samples every 2-weeks post-injection. **d** Blood cell populations in blood samples collected at 12-weeks post-injection. **e** White blood cell (WBC) and **f** platelet count in blood samples collected every 4-weeks post-injection. Quantification of (**g**) ALT (U/L, unit per liter) and (**h**) HDL in plasma samples collected every 2-weeks post-injection. Bars indicate mean ± SD (*n* = 3-4) and statistical significance was calculated using two-way ANOVA with Tukey’s test (*p < 0.033; **p < 0.002; ***p < 0.0002). Exact *p* value is indicated in Supplementary Data 1.

As disease model, we used a *Lipa*^*−/−*^ mouse model generated by deleting exon 4 of *LIPA* gene, with virtually no residual LAL expression or activity (Supplementary Fig. 1). *Lipa*^*−/−*^ mice failed to thrive and remained smaller compared to WT mice. To deliver/express the human *LIPA* cDNA we took advantage of the recombinant single-stranded adeno associated viral vector (rAAV) 8 and the hepatocyte-specific human α1-antitrypsin (hAAT) promoter (rAAV8.hAAT.LIPA), a capsid/promoter combination already described in multiple preclinical studies^24–28^. This rAAV8 construct was intravenously administered into 10–12-week-old male *Lipa*^*−/−*^ mice at 4 different doses: 5 × 10^11^ viral genome per kilogram (vg/kg) (low dose (LD) 1), 1 × 10^12^ vg/kg (LD2), 3 × 10^12^ vg/kg (middle dose; MD), 1x10^13^ vg/kg (high dose; HD). Body weight, LAL activity, haematological parameters, hepatic transaminase (ALT, AST; liver damage markers) and high-density lipoprotein (HDL)-cholesterol concentration were monitored every other week for 12-weeks post-administration (Fig. 1a). Soon after injection, we observed that treated mice gained body weight according to the injected rAAV8 dose compared to *Lipa*^*−/−*^ mice (Fig. 1b). Since our approach depends on the hepatic expression and secretion of therapeutic LAL enzyme in the circulation for systemic cross-correction, we measured the LAL activity in plasma where it is normally absent. As expected, LAL activity was significantly increased 2-weeks post-injection and positively correlated with the injected rAAV8 dose, reaching up to 4-fold higher activity in circulation compared to WT mice (Fig. 1c). Noteworthy, LD1 did not result in any increase of LAL activity, while LD2 reached a plateau at 8-weeks post injection. Some LAL activity was still observed in *Lipa*^*−/−*^ mice, probably due to unspecific signals from the high abundance of other lipases in plasma.

Next, we monitored haematological parameters at 4-, 8- and 12-weeks post injection. In *Lipa*^*−/−*^ mice, we observed a shift in blood cell composition (Fig. 1d; Supplementary Fig. 2a), with a significant increase in white blood cells (WBCs) (Fig. 1e), as previously described^29,30^. This inflammatory profile was confirmed by thrombocytosis (Fig. 1f) with larger platelets (Supplementary Fig. 2b) and microcytic anemia (Supplementary Fig. 2c, e). In addition, we observed an increase in AST and ALT concentrations (Fig. 1g; Supplementary Fig. 2f), indicating liver damage, and a decrease in HDL (Fig. 1h), a major risk factor for cardiovascular disease. Interestingly, all parameters were fully corrected within 2-weeks post-injection with all rAAV8 doses, except for LD1, which showed a partial rescue.

### Hepatic transduction restores LAL expression in a dose dependent manner and normalizes hepatosplenomegaly

**Fig. 2:**
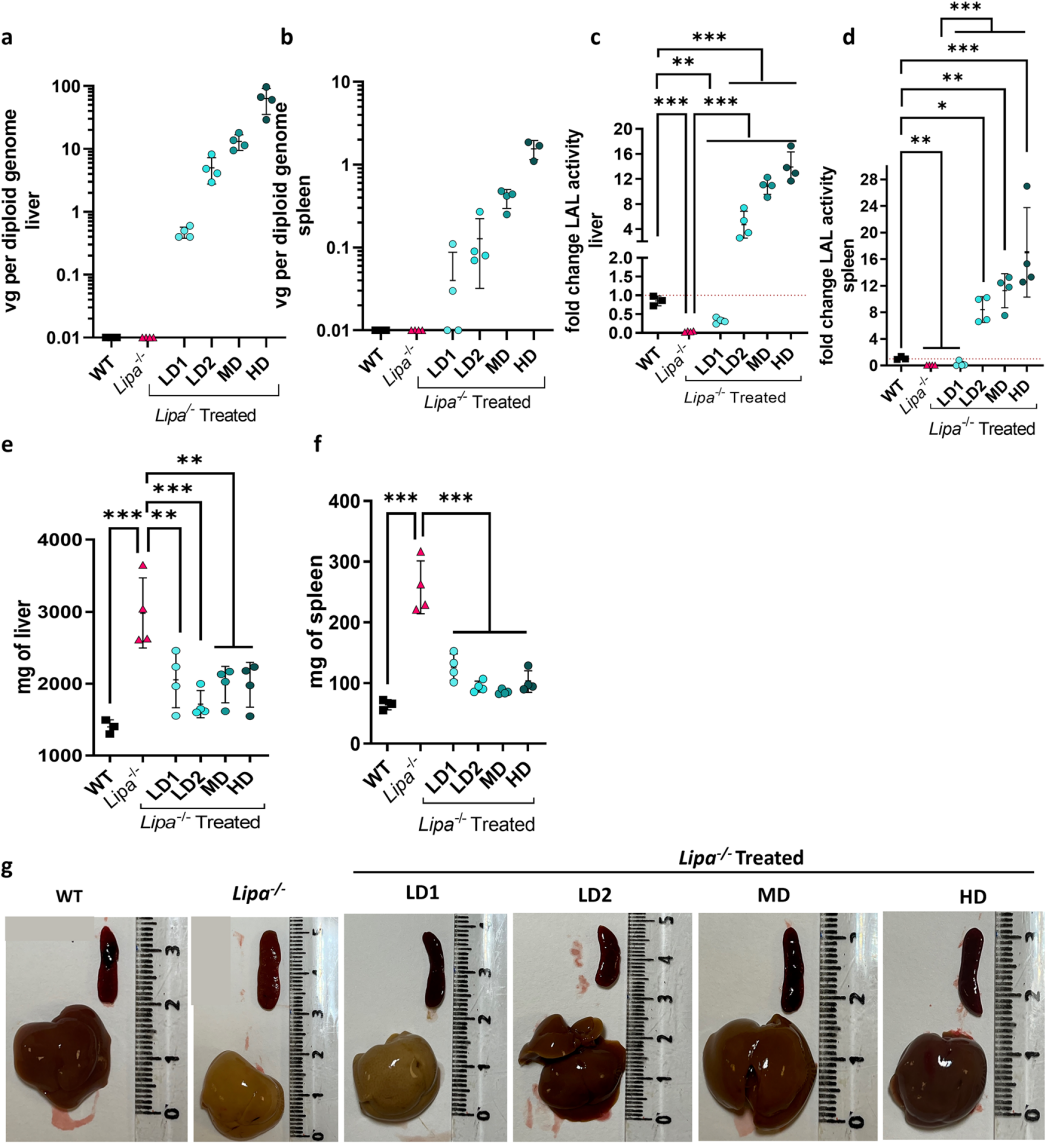
rAAV8 transduction of liver and spleen of *Lipa*^*−/−*^ mice increases LAL activity and corrects hepatosplenomegaly. rAAV8 transduction level for different vector doses at 12-weeks post injection (**a**) in liver and (**b**) spleen. vg vector genome. Fold change of LAL activity over WT mice (**c**) in liver and (**d**) spleen for different vector doses. Weight of (**e**) liver and (**f**) spleen at 12-weeks post-injection for different vector doses. **g** Representative images of liver and spleen at 12-weeks post-injection. Ruler indicates centimetres. Bars indicate mean ± SD (*n* = 3-4) and statistical significance was calculated using two-way ANOVA with Tukey’s test (*p < 0.033; **p < 0.002; ***p < 0.0002). Exact *p* value is indicated in Supplementary Data 1.

Twelve-weeks after rAAV8 injection, the mice were sacrificed to evaluate the transduction efficiency, LAL enzymatic activity and hepatosplenomegaly. As expected for the rAAV8 serotype, vector genomes were higher in the liver than in the spleen and jejunum, and they were proportional to the injected rAAV8 dose (Fig. 2a, b; Supplementary Fig. 3a). The same trend was observed for LAL enzymatic activity (Fig. 2c, d; Supplementary Fig. 3b), with the lowest hepatic transduction rate (LD1; 0.5 + /− 0.1 copy per diploid genome, mean + SD) resulting in a LAL activity around 35% of WT level (Fig. 2c). Noteworthy, the LAL activity observed in the spleen and the jejunum could be the combined result of both rAAV8 transduction and cross-correction from hepatocyte-secreted LAL into the circulation. We then assessed the efficacy of LAL in improving the affected organs. Hepatosplenomegaly (Fig. 2e, g) and liver discoloration (Fig. 2g) due to increased lipid accumulation in *Lipa*^*−/−*^ mice were significantly decreased with each of the four rAAV8 doses tested (Fig. 2e, g). Interestingly, the LD1 reduced organs weight, although liver discoloration was only partially corrected at 12-weeks post-administration (Fig. 2g).

Overall, these findings demonstrate that rAAV8 injection efficiently transduced the liver and corrected hepatosplenomegaly.

### LAL overexpression restores lipid homeostasis in liver and spleen

**Fig. 3:**
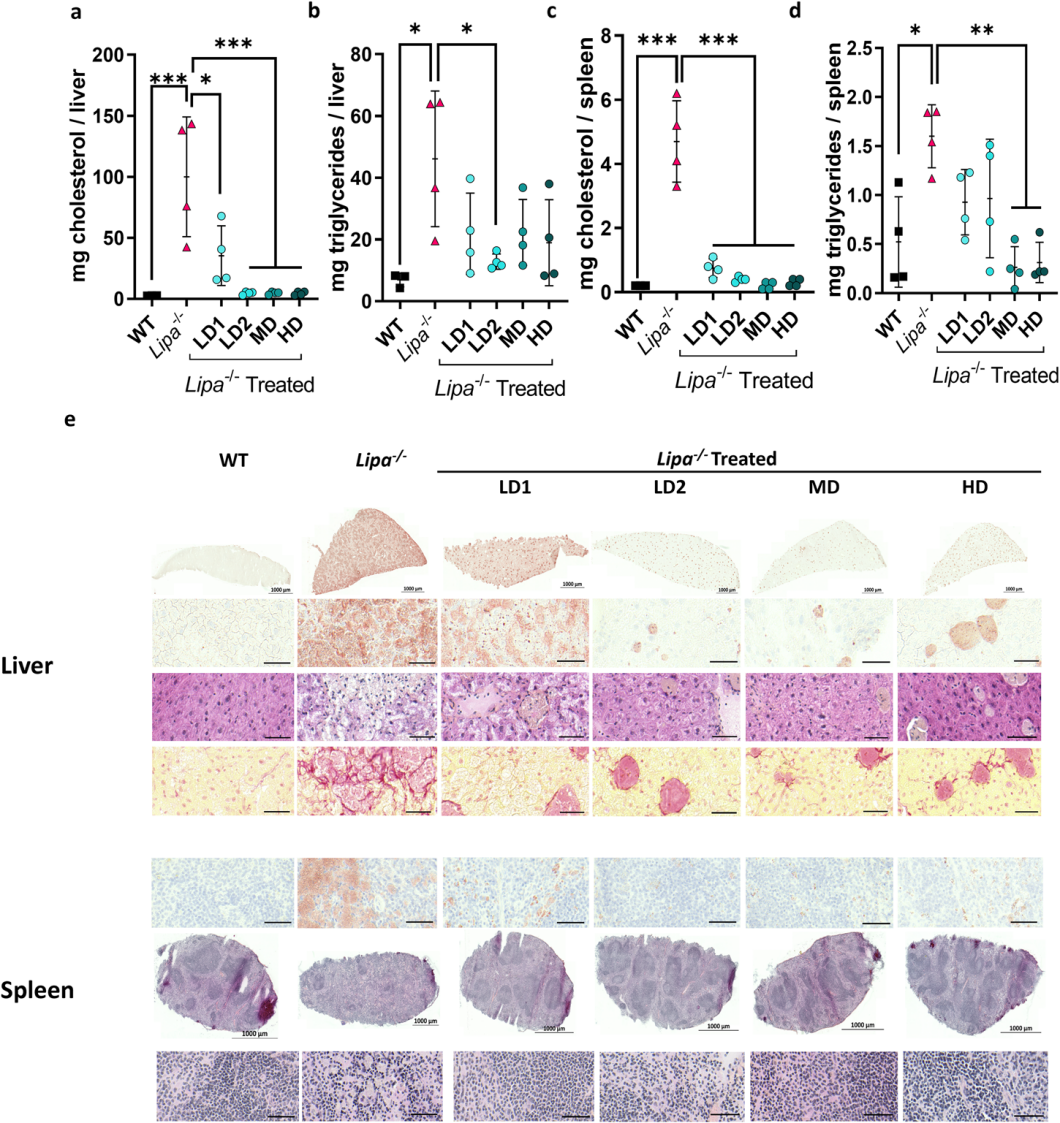
rAAV8 administration corrects lipid accumulation in liver and spleen. Quantification of cholesterol and triglycerides in (**a**, **b**) liver and (**c**, **d**) spleen at 12-weeks post-injection. mg = milligrams. Bars indicate mean ± SD (*n* = 3-4) and statistical significance was calculated using one-way ANOVA with Tukey’s test (*p < 0.033; **p < 0.002; ***p < 0.0001). Exact *p* value is indicated in Supplementary Data 1. **e** Histological sections: Oil red O (ORO), Haematoxylin Eosin Saffron (HES) and Sirius red (SR) staining of liver (top panel), spleen (bottom panel) at 12-weeks post-injection (scale bar: 50 µm unless differently noted).

In *Lipa*^−/−^ mice, hepatosplenomegaly is caused by massive accumulation of total cholesterol and triglycerides (Fig. 3a, d; Supplementary Fig. 3c, f). Twelve-weeks post-treatment, all rAAV8 doses fully reverted cholesterol and triglyceride content in the liver of *Lipa*^−/−^ mice, with the exception of LD1, which reduced cholesterol deposits by more than half (Fig. 3a; Supplementary Fig. 3c). Similar results were obtained for the spleen, although lipid accumulation was less pronounced than in the liver (Fig. 3c, d; Supplementary Fig. 3e, f). In the jejunum, we observed only cholesterol accumulation, which was corrected upon rAAV8 treatment (Supplementary Fig. 3g, h).

We then performed liver and spleen histological analyses to assess architectural alterations. Oil red O (ORO) staining revealed pronounced accumulations of neutral lipids in *Lipa*^−/−^ mice (Fig. 3e; Supplementary Fig. 3i, j), while haematoxylin and eosin staining revealed an increase in connective tissue and a loss of red and white pulps in spleen (Fig. 3e). Finally, appearance of fibrosis was observed by Sirius red staining (Fig. 3e; Supplementary Fig. 3k). All these pathological signs were mostly reversed 12-weeks after rAAV8 injection, except for LD1 that showed partial efficacy.

### rAAV8 injection results in improved survival rate and disease correction up to 34-weeks post-treatment

In light of recent Crigler–Najjar clinical data suggesting that the minimal therapeutically effective dose in mice did not yield optimal results in humans^24,25^, we chose to assess MD—over LD2—at longer time points to balance effective LAL expression while minimizing potential rAAV-related toxicity.

Hence, we injected *Lipa*^*−/−*^ mice and biweekly monitored body weight, LAL activity, haematological parameters, transaminases, and HDL-cholesterol for 34-weeks post-injection and collected tissues and organs at 4-, 12- and 34-weeks post-injection.

**Fig. 4:**
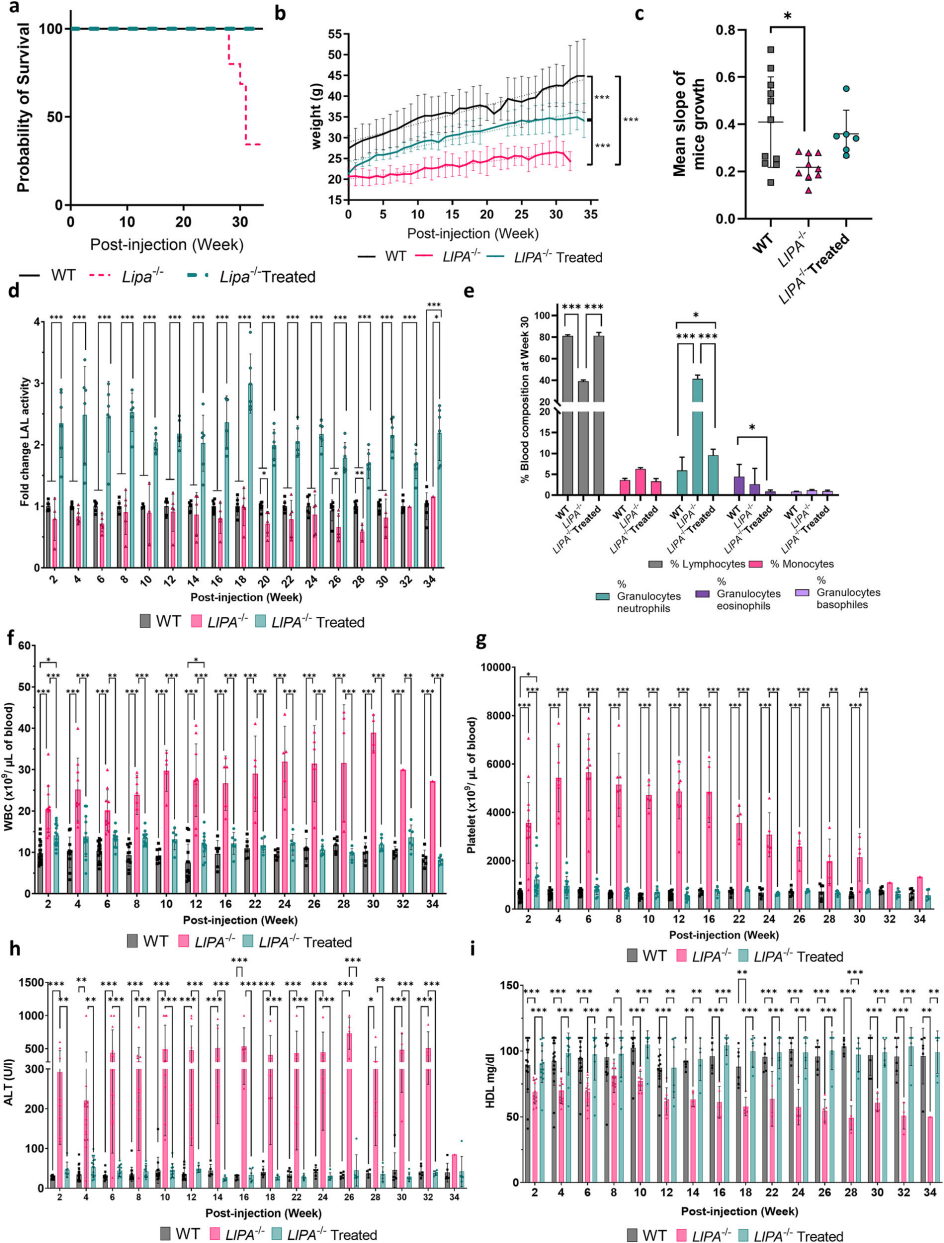
rAAV8 administration increases survival and body weight of male *Lipa*^−/−^ mice and corrects haematological parameters and dyslipidaemia for 34-weeks post-injection. **a** Percentage of mice survival. **b** Mice weight (gr: grams) was recorded every week (*n* = 6-11). **c** Linear slopes of mice weight. Bars indicate mean ± SD (*n* = 3-4) and statistical significance was calculated using one-way ANOVA with Turkey’s Test (*p < 0.033; **p < 0.002; ***p < 0.0002). Exact *p* value is indicated in Supplementary Data 1. **d** Fold change of LAL activity over WT mice measured in plasma samples every 2-weeks post-injection (*n* = 3-11). **e** Blood cell populations in blood samples collected at 30-weeks post-injection (*n* = 3-6). **f** White blood cell (WBC) and (**g**) platelet counts in blood samples collected every 2-weeks post-injection (*n* = 1-20). Quantification of (**h**) ALT (U/L, unit per liter) and (**i**) HDL in plasma samples collected every 2-weeks post-injection (*n* = 1-20). Bars indicate mean ± SD and statistical significance was calculated using two-way ANOVA with Tukey’s test (*p < 0.033; **p < 0.002; ***p < 0.0002). Exact *p* value is indicated in Supplementary Data 1.

Our data demonstrated that rAAV8 administration prolonged survival, improved body weight, and normalized growth of *Lipa*^−/−^ mice for at least 34-weeks post-injection (Fig. 4a, c). In addition, LAL activity in plasma was 2.5-fold increased (Fig. 4d), which fully corrected blood cell composition (Fig. 4e; Supplementary Fig. 4a), WBC counts (Fig. 4f), thrombocytosis (Fig. 4g; Supplementary Fig. 4b), and microcytic anemia (Supplementary Fig. 4c, e). Finally, LAL expression reduced ALT (Fig. 4h) and normalized HDL levels (Fig. 4i). Noteworthy, correction of haematological and serum parameters was already completed after 2-week post-injection and remained stable throughout the entire 34-weeks follow-up period.

**Fig. 5:**
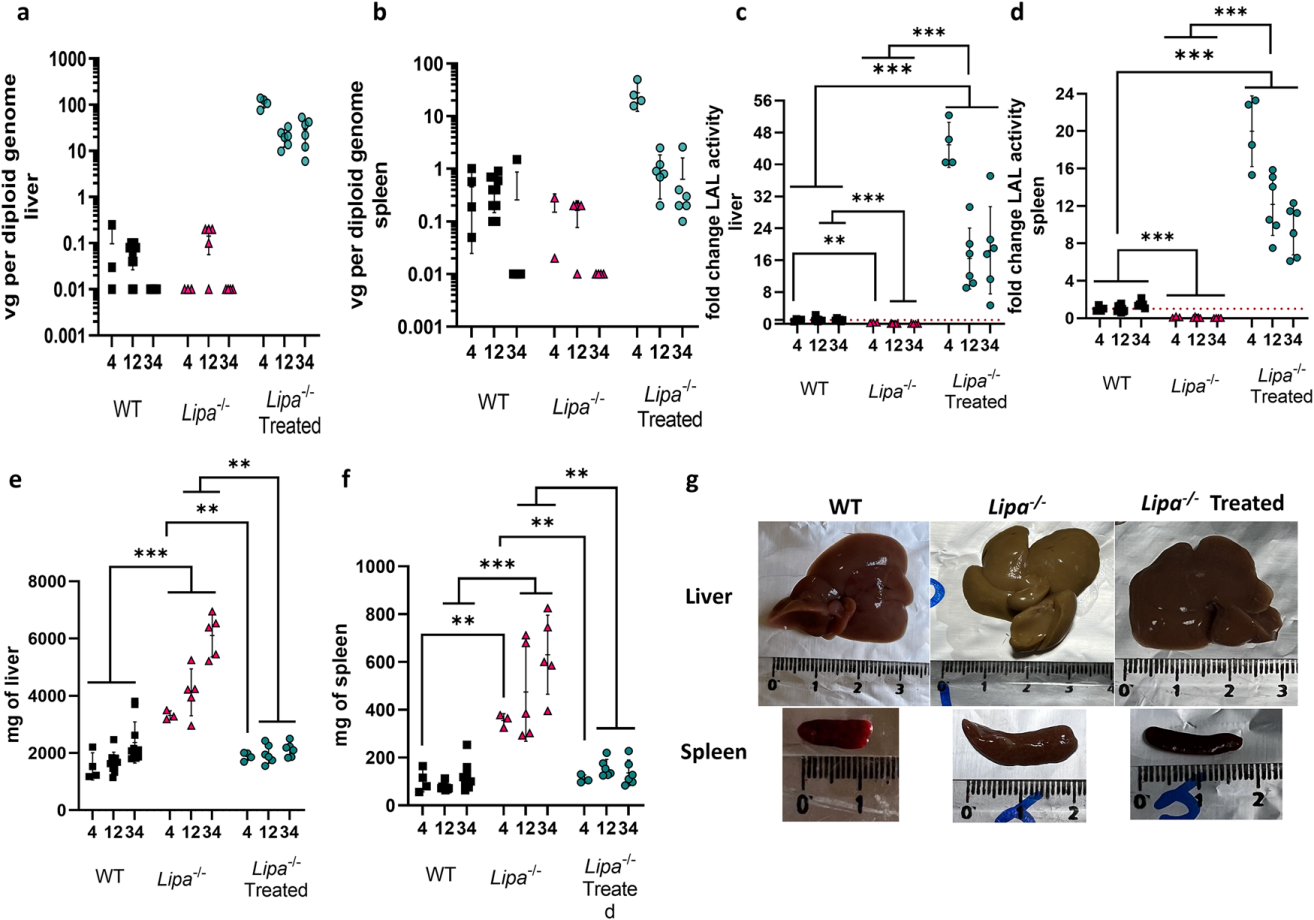
rAAV8 transduction of liver and spleen of *Lipa*^−/−^ mice results in long-term increase of LAL activity and correction of hepatosplenomegaly. rAAV8 transduction level in (**a**) liver and (**b**) spleen at indicated time points (weeks) (*n* = 4-10). vg vector genome. Fold change of LAL activity over WT mice in (**c**) liver and (**d**) spleen at indicated time points (weeks). Weight of (**e**) liver and (**f**) spleen at indicated time points (weeks). **g** Representative images of liver and spleen at 12-weeks post-injection. Bars indicate mean ± SD (*n* = 4-10) and statistical significance was calculated using two-way ANOVA with Tukey’s test (*p < 0.033; **p < 0.002; ***p < 0.0002). Exact *p* value is indicated in Supplementary Data 1.

**Fig. 6:**
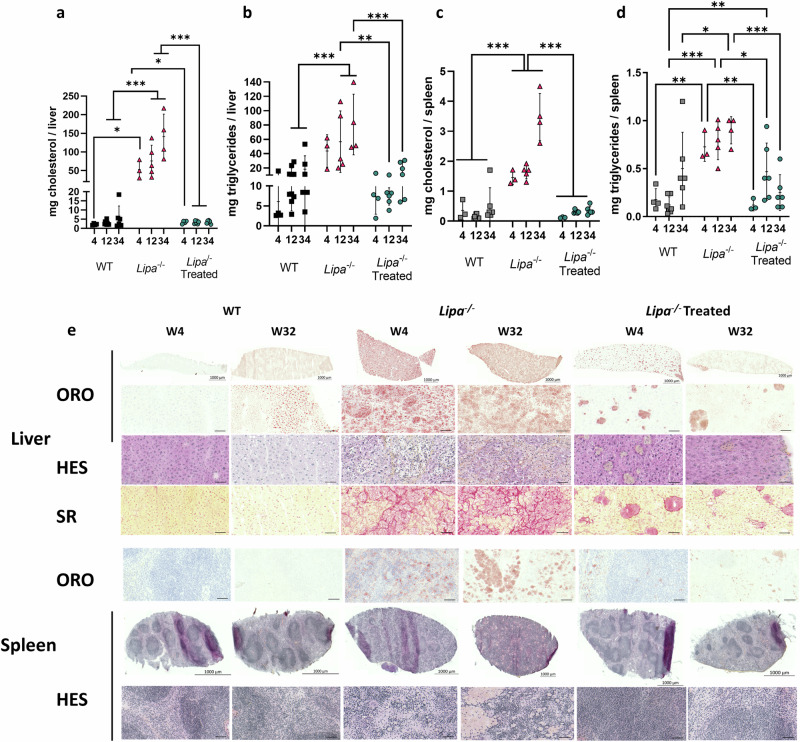
rAAV8 administration corrects lipid accumulation in liver and spleen long-term. Quantification of cholesterol and triglycerides in (**a, b**) liver and (**c, d**) spleen at indicated time points (weeks). Bars indicate mean ± SD (*n* = 4-10). Statistical significance was calculated using two-way ANOVA with Tukey’s test (*p < 0.033; **p < 0.002; ***p < 0.0001). Exact *p* value is indicated in Supplementary Data 1. **e** Histological sections with: Oil red O (ORO; top row), Haematoxylin Eosin Saffron (HES; middle row) and Sirius red (SR; bottom row) staining of liver (top panel), and spleen (bottom panel) at 4- and 34-weeks (scale: 50 µm).

Treated mice were sacrificed at 4-, 12- and 34-weeks post-injection to evaluate rAAV8 transduction efficiency, LAL activity in organs and hepatosplenomegaly over time. As observed previously, we detected higher rAAV8 transduction and LAL expression in the liver compared to the spleen and jejunum at all-time points (Fig. 5a–d; Supplementary Fig. 5a, b). In *Lipa*^−/−^ mice, the liver weight accounted for one-fourth of the total body weight, which was reduced to one-twentieth of the total body weight in rAAV8-treated, as in WT mice (Fig. 5e, g). The enlargement of both the liver and the spleen (Fig. 5f) was caused by the accumulation of total cholesterol and triglycerides, as quantified biochemically (Fig. 6a, d; Supplementary Fig. 5c, f) and histologically by ORO staining (Fig. 6e; Supplementary Fig. 5g, h). In addition, SR staining revealed architectural alterations and fibrosis in the liver (Fig. 6e; Supplementary Fig. 5i). To a lower extent, lipid accumulation and structural alterations were also observed in the jejunum (Supplementary Fig. 5j, m) and thymus (Supplementary Fig. 6). Noteworthy, organ correction was already visible after 4-weeks post-injection and for at least 34-weeks.

### rAAV8 injection improves general metabolic, mitochondrial, and immune system dysregulation

**Fig. 7:**
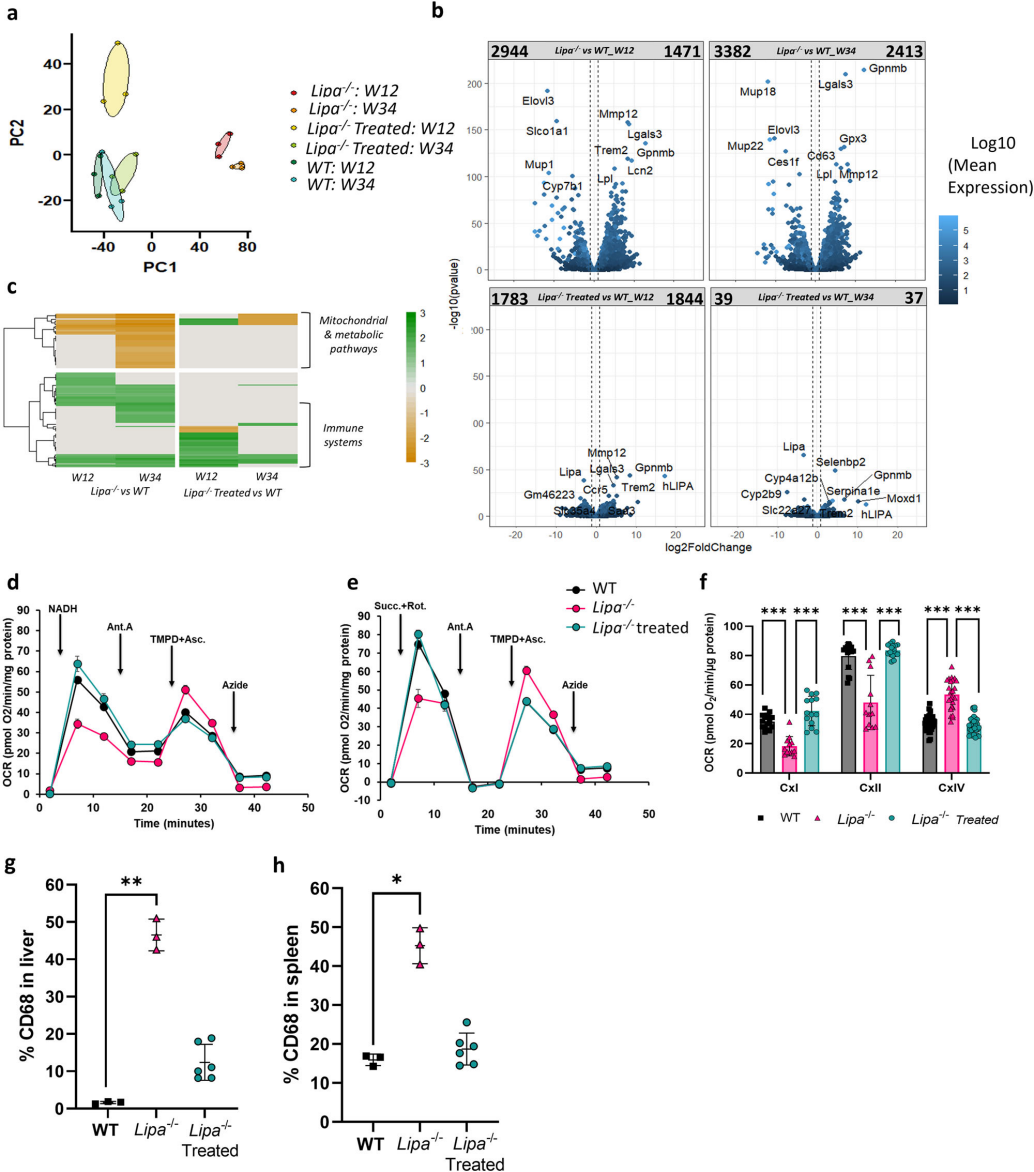
rAAV8 administration corrects expression of gene related to metabolic, immune, and mitochondria. Differential mRNA expression analysis was performed on liver WT, *Lipa*^*−/−*^ and rAAV8-treated *Lipa*^*−/−*^ mice. **a** Principal component analysis (PCA) visualizes the separation between experimental groups. **b** Volcano plots display genes according to log2 fold change and -log10 *P* value. Colour shade indicates the log10 mean expression level of all analysed animals (*n* = 3). For some dots the associated gene name is indicated. On top of each plot, the names indicate the samples compared, while the numbers indicate how many genes (dots) are present in each quadrant. Vertical dotted lines indicate ± 2-fold change. **c** Gene ontology analysis by Gene Set Enrichment Analysis (GSEA). Colour shades indicate normalized enrichment score (NES) of the indicated mice group comparison at 12- and 34-weeks post-injection. **d, e** Representative traces of the oxygen consumption rates (OCRs) in liver of WT, *Lipa*^*−/−*^ and rAAV8-treated *Lipa*^*−/−*^ mice at 34-weeks post-treatment. Where indicated, the following compounds were injected into the assay micro-chambers: NADH, Rotenone+Antimycin A (Rot+Ant A), Succinate+Rotenone (Succ+Rot), Antimycin A (AntA), Asc/TMPD (ascorbate+ TMPD), Azide. **f** Metabolic parameters inferred from the OCR assays and corrected for residual activity in the presence of the respiratory chain inhibitors for mitochondria isolated from mouse at 34- weeks post injection. Bars indicate mean ± SD (*n* = 3-4 with 3 or 4 biological replicates for each group) and statistical significance was calculated using two-way ANOVA with Bonferroni post-hoc analysis (*p < 0.033; **p < 0.002; ***p < 0.0002). Exact *p* value is indicated in Supplementary Data 1. % of CD68-stained area per section in the liver (**g**) and spleen (**h**) at 34-weeks post-treatment. Bars indicate mean ± SD (*n* = 3-6) and statistical significance was calculated using one-way ANOVA with Kruskal-Wallis Test (*p < 0.033; **p < 0.002; ***p < 0.0002). Exact *p* value is indicated in Supplementary Data 1.

Metabolic alterations can have profound and far-reaching effects beyond lipid accumulations. Therefore, to gain a comprehensive understanding, we performed an unbiased transcriptomic analysis of WT, *Lipa*^−/−^ and rAAV8.hAAT.LIPA-treated *Lipa*^−/−^ mouse livers at 12- and 34-weeks post-injection. Principal component analysis (PCA) of gene expression showed that: 1) WT mice at 12- and 34-weeks grouped together, similarly to *Lipa*^−/−^ mice at 12 and 34-week; 2) *Lipa*^−/−^ mice clearly diverged from the other groups at both time points; 3) rAAV8-treated *Lipa*^−/−^ mice diverged from *Lipa*^−/−^ mice and grouped with WT mice at 34-weeks (Fig. 7a).

Differential gene expression analysis revealed that ~4400 and ~5800 genes changed between WT and *Lipa*^−/−^ mice at 12- and 34-weeks, respectively (Fig. 7b top row). Upon rAAV8 mediated h*LIPA* expression (Supplementary Fig. 7a), both the number and the fold change of dysregulated genes was reduced, with only 76 genes differing between treated *Lipa*^−/−^ and WT mice at 34-weeks post-injection (Fig. 7b bottom row).

Gene ontology analysis of differentially expressed genes between WT and *Lipa*^−/−^ mice identified two highly dysregulated pathways: (i) the downregulation of the metabolic and mitochondrial pathways in *Lipa*^−/−^ mice; (ii) the upregulation of the immune systems in *Lipa*^−/−^ mice (Fig. 7c; left panel). rAAV8 administration normalized gene expression related to metabolic pathways at 12- and 34-weeks, while gene associated with the immune system were normalized only at 34-weeks (Fig. 7c; right panel).

Nowadays, the crosstalk between mitochondria and lysosomes is well known in normal as well as pathological conditions, such as lysosomal storage disorders^31–33^. For WD, dysfunctional mitochondria have been recently described in skeletal muscles of *Lipa*^−/−^ mice^34^; however, their involvement is still not clear for liver^35^. We isolated mitochondria from WT, *Lipa*^−/−^ and rAAV8-treated *Lipa*^−/−^ frozen mouse liver (at 12- and 34-weeks post-injection) and measured mitochondrial oxidative function. In particular, we assessed the efficiency of the electron transport chain complexes working together by exogenous substrates supplying electrons to complex I (NADH), II (succinate) and IV (ascorbate + N,N,N’,N’-tetramethyl-p-phenylenediamine dihydrochloride (TMPD)) (Fig. 7d, e). The oxygen consumption rates (OCR) elicited by complex I and II substrates (NADH and succinate respectively) were significantly reduced in *Lipa*^−/−^ mitochondria but completely restored upon rAAV8 administration already 12-weeks post-injection (Supplementary Fig. 7b, d) and until 34-weeks post-injection (Fig. 7f). Surprisingly, the addition of complex IV substrates (ascorbate + TMPD) revealed a different scenario, with *Lipa*^−/−^ mitochondria showing a significant increase of respiratory activity compared to WT and rAAV8-treated *Lipa*^−/−^ mitochondria, probably due to a compensatory effect^36,37^.

Altogether, these results suggest a reduced mitochondrial respiratory capacity of *Lipa*^−/−^ mitochondria, which was fully corrected by rAAV8 treatment.

To further elucidate the immune infiltration, we labelled and quantified the percentage of CD68 positive area in liver (Fig. 7g; Supplementary Fig. 7e), spleen (Fig. 7h; Supplementary Fig. 7e), and jejunum (Supplementary Fig. 7e, f) sections and observed a strong reduction of inflammation in all tissues upon treatment at 34-week post-injection.

Overall, administration of 3 x 10^12^ vg/kg of rAAV8 was sufficient to stably and completely correct the pathological phenotype of the *Lipa*^−/−^ mouse model for 34-weeks post-injection.

## Discussion

In this study, we evaluated a liver directed gene therapy for LAL-D in a *Lipa*^−/−^ mouse model that displays reduced lifespan, failure to thrive and severe hepatosplenomegaly associated with lipid accumulation. By using a single-stranded rAAV8 encoding for the human *LIPA* cDNA under the control of the hepatocyte specific human α1-antitrypsin promoter, we demonstrated that transduced hepatocytes could express and secrete LAL, which retains its enzymatic activity and systemically cross-corrects LAL-D metabolic defect. In addition, we established the minimal rAAV8 vector dose required to extend mice survival, restore lipid homeostasis, and improve transcriptomic imbalance and mitochondrial activity.

We intravenously injected *Lipa*^−/−^ mice with four different rAAV8 doses and observed a transduction level and LAL activity proportional to the injected dose, suggesting that LAL overexpression does not result in liver toxicity. Secreted LAL was able to cross-correct blood cells and restore haematological parameters. In addition, expressed LAL ameliorated mouse weight and growth rate, corrected hepatosplenomegaly, and reduced lipid accumulation in the jejunum. Interestingly, even the lowest rAAV8 dose (5 x 10^11^ vg/kg) significantly reduced lipid accumulation and decreased disease burden. At high dose (1 x 10^13^ vg/kg), instead, we observed a plateau in LAL expression, which could be explained by saturation of the machinery involved in LAL synthesis or rAAV transduction of cells other than hepatocytes, where the hAAT promoter might be less active.

We then performed a more detailed study using the minimal rAAV8 dose (3 x 10^12^ vg/kg) that improved lifespan and ameliorated both mouse weight and growth rate up to 34-week post-treatment. In particular, we evaluated short-term (4-weeks), middle term (12-weeks) and long-term (34-weeks) disease correction to confirm the efficacy and safety of our gene therapy treatment over time. Although we observed efficient hepatocyte transduction and LAL expression at all time points, there was a decrease between 4- and 12-weeks. Since rAAV genome remains mainly episomal, cellular proliferation due to the underlying liver disease could more likely explain this reduction, as previously reported for liver growth in young mice^38^. In addition, we cannot exclude the possibility of an immune response against the transduced hepatocytes^39^, potentially enhanced by the disease pre-existing inflammation. Finally, reduced LAL expression could be due to a translational shutdown of transgene expression as seen for FVIII^40^. Additional studies of hepatocyte proliferation and immune inflammation will be performed to clarify the underlying mechanism. Interestingly, LAL expression fully corrected haematological parameters, dyslipidaemia and hepatic transaminase from 2-weeks post-injection and lipid storage and tissue architecture of liver, spleen, jejunum, and thymus from 4-weeks post-injection. Few small lipid droplets remained visible in the liver at late time points possibly due to transduction zonation^41^ or reduced rAAV8 transduction in area of pre-existing liver injury in *Lipa*^−/−^ mice, e.g. fibrosis^42^. Finally, by liver transcriptomic analysis and mitochondrial respirometry we observed a dysregulation of metabolic and immune system pathways^43^ and an impairment in mitochondrial activity, which were fully normalized at 34-weeks post-injection. Overall, our in vivo gene therapy strategy appears to be an effective approach to achieve stable LAL expression to correct LAL-D phenotype in a relevant *Lipa*^*−/−*^ mouse model.

The current standard of treatment for patients affected by LAL-D is life-long weekly administration of recombinant human LAL enzyme (Sebelipase alfa, Kanuma) to reduce lipid storage and alleviate disease symptoms^44^. Although effective in managing the condition, this treatment is not curative, and its effectiveness varies. Furthermore, it does not completely normalize all clinical manifestations, such as abdominal distortion, hepatosplenomegaly, and lipid accumulation in lymph nodes and the digestive wall^45^. In addition, the repetitive administration of ERT may lead to the development of neutralising antibodies against the therapeutic enzyme, diminishing its efficacy over time^46^. The described rAAV8 gene therapy has the potential to be a one-time curative treatment that maintains a constant level of therapeutic proteins and induces immune tolerance to the expressed transgene^47,48^ or modulates an already existing immune response^49–51^. Furthermore, hepatocyte-expressed LAL should result in enhanced pharmacokinetics and uptake of the enzyme in peripheral tissues compared to ERT, for which differential organ response is observed^52,53^, and undergo endogenous post-translational modifications (e.g., glycosylation), which could reduce the development of neutralizing antibodies^54^.

Lam et al.^55^ recently described a liver directed gene therapy approach based on a recombinant self-complementary AAV vector serotype rh74 (rscAAVh74) encoding *LIPA* gene under the control of the ubiquitous mini cytomegalovirus promoter. Administration of the vector to *Lipa*^−/−^ mice successfully corrected the LAL-D phenotype by decreasing hepatosplenomegaly, dyslipidaemia, liver damage, and lipid accumulation. Although promising, this study has several limitations that requires further investigations. The authors used the rAAVh74 serotype, which has been selected to transduce muscles^56–58^ and to treat muscular disorders (Dysferlinopathies: NCT02710500, Duchenne Muscular Dystrophy: NCT02704325, NCT02376816, NCT03375164). In addition, they used a self-complementary rAAV, which may increase the innate immune response compared to natural single-stranded rAAV vectors^59–61^. Finally, to drive the expression of the LAL transgene they used the viral CMV promoter, which may become inactive due to DNA methylation^62,63^. As an alternative, we propose using a single stranded rAAV8 to deliver the *LIPA* cDNA under the control of a liver-specific promoter. The rAAV8 serotype is currently the gold-standard for liver targeting and has been successfully tested in clinical trials for various metabolic disorders, such as glycogen storage disease type 1a (NCT03517085), Crigler Najjar (NCT03466463), mucopolysaccharidosis type VI (MPSVI) (NCT03173521), and Pompe disease (NCT03533673 and NCT04093349). It is worth noting that our rAAV8 corrective dose was ~10 times lower than the previously suggested dose, reducing the risk of patient toxicity^64^. Regarding the mouse model, the authors used a *Lipa*^−/−^ mouse model with normal body weight and growth rate and did not perform any survival analysis^55^. Our *Lipa*^−/−^ mouse model, on a different genetic background and with a different LIPA mutation, allowed us to assess rAAV8-mediated correction of body weight, growth rate and lifespan. In addition, we conducted a follow-up analysis for up to 34-weeks post-injection and assessed biweekly several haematological parameters as a simple method to evaluate disease progression and gene therapy correction over time. Finally, using liver transcriptome analysis, we demonstrated a reduction in inflammation and correction of metabolic and mitochondrial pathways. To assess whether the mitochondrial alterations detected by transcriptomic analysis resulted in functional modifications, we measured the respiratory capacity of mitochondria. As reported in skeletal muscle fibers of *Lipa*^−/−^ mice^34^ and in line with other lysosomal storage disorders^65^, we found a significant decline of mitochondrial respiratory fluxes in mitochondria from *Lipa*^−/−^ livers. The respiratory fluxes were reduced when using Complex-I or Complex-II-dependent substrates, whereas they were increased when using exogenous substrates supplying electrons to Complex IV, suggesting a crucial role for Complex IV in compensating energy demand^36,37^. Interestingly, administration of rAAV8 to *Lipa*^−/−^ mice fully restored the mitochondrial defect, both 12- and 34-weeks post-injection, confirming the effectiveness of our proposed therapy.

This in vivo gene therapy approach may still be hampered by preexisting neutralizing antibodies and cell-mediated immune responses against rAAV8 vectors with a 20–30% prevalence^66,67^. A possible solution could be Imlifidase, a protease able to cleave IgG, that was shown to reduce pre-existing neutralizing antibody reactions against rAAV8 capsids in non-human primates, thus allowing vector re-administration^68^. In addition, novel rAAV serotypes could be tested to reduce immunogenicity and increase liver targeting specificity^69,70^, potentially reducing the required rAAV dose. To further decrease the rAAV dose and maximize therapeutic benefits, we will optimise codon usage and change the signal peptide sequence of the *LIPA* cDNA to improve LAL expression and secretion^71^.

Although this rAAV8 strategy would be effective in patients with CESD, its efficacy would be limited in infants with WD, where episomal rAAV genome would be progressively diluted and lost upon liver growth^38,72^. Therefore, a clinical translation will require treating patients with ERT until they reach the minimal age for rAAV treatment (5 years)^73^, similarly to what has recently been described for allogenic hematopoietic stem cell transplantation in LAL-D^8,74^. In addition, research on haemophilia showed stable expression of therapeutic protein for up to 10-years using rAAV8 in human^75^ and dog^76^, possibly due to random genomic integration of rAAV DNA^77^. Following this observation, we will investigate the use of DNA nucleases to achieve stable liver transgene targeted integration in a safe and highly transcribed genomic locus^78–84^. Since a >2% increase in LAL enzyme function could significantly improve clinical condition, we will need to edit a small fraction of hepatocytes, <10% according to other diseases^78–84^.

For treating both WD infants and adults, a possible alternative to rAAV would be a LAL cDNA gene addition strategy in autologous hematopoietic stem cells, which guarantees stable transgene integration and expression over time, as already shown for other lysosomal storage disorders^85–88^.

Finally, before moving to a clinical trial, we plan to treat the Donryu rat model for LAL-D that displays a severe disease phenotype closer to that of WD patients and has a life expectancy of 120 days^89^ and carefully assess potential side-effects of the treatment, such as long-term LAL overexpression and rAAV random genomic integration.

In conclusion, our data suggest that a liver directed rAAV8 gene therapy strategy may be an effective curative treatment for LAL-D.

## Supplementary information

Supplemental material

Description of Additional Supplementary File

Supplementary Data 1

reporting-summary

## Data Availability

The source data and statistical analysis for all figures, are accessible from the Supplementary Data 1 file. The RNA-seq data, both raw and processed, are available on the GEO data set: GSE252742.
