# Supplementary material for: Rescue of lysosomal acid lipase deficiency in mice by rAAV8 liver gene transfer
Source: Commun Med (Lond). 2025 Apr 11;5:110. doi: 10.1038/s43856-025-00816-8 (PMC11992068; doi:10.1038/s43856-025-00816-8)
Supplement: Supplementary file 1 — Supplemental material [file 43856_2025_816_MOESM1_ESM.pdf]

Table of supplemental figures:

Supplementary Fig. 1. Description of 13-week-old *Lipa*$^{-/-}$ mouse model. ______________ 2

Supplementary Fig. 2. Blood analysis of *Lipa*$^{-/-}$ mice treated with 4 different rAAV8 doses. ______________ 4

Supplementary Fig. 3. Quantification of cholesterol and triglycerides per mg of liver, spleen, and jejunum 12-weeks post-injection. ______________ 6

Supplementary Fig. 4. Long-term blood analysis of rAAV8.hAAT.LIPA treated *Lipa*$^{-/-}$ mice. ______________ 8

Supplementary Fig. 5. Quantification of cholesterol and triglycerides per mg of liver, spleen, and jejunum. ______________ 10

Supplementary Fig. 6. Quantification of lipid deposits in thymus ______________ 12

Supplementary Fig. 7. *LIPA* expressed upon rAAV8 administration corrects mitochondrial impairment in liver and immune infiltration. ______________ 14

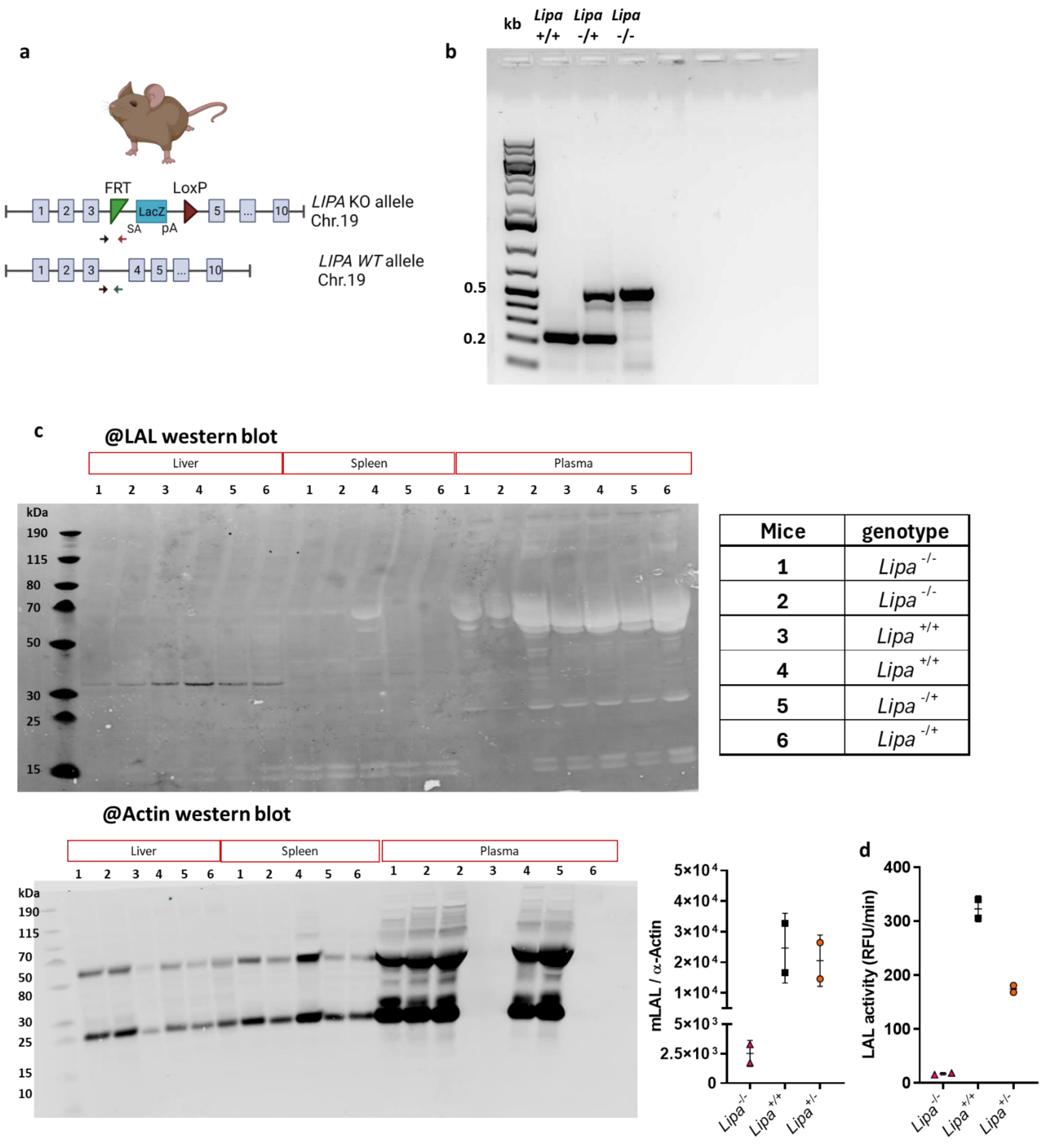

Supplementary Fig. 1. Description of 13-week-old *Lipa*$^{-/-}$ mouse model. **a** *LIPA* gene in KO and WT alleles. Arrows represent genotyping primers (black: common LAL F, red: LAL-KO R and blue: LAL-WT R; Table 1). **b** Agarose gel of the genotyping PCR. Kb represents the 1kb DNA ladder. **c** Western blot of mouse liver with mLAL antibody and mActin antibody and its

quantification relative to α-actin expression. **d** Relative fluorescence units (*RFU*) of LAL enzymatic activity in mouse liver. Bars indicate mean ± SD (n=2). Raw data are available in Supplementary Data 1.

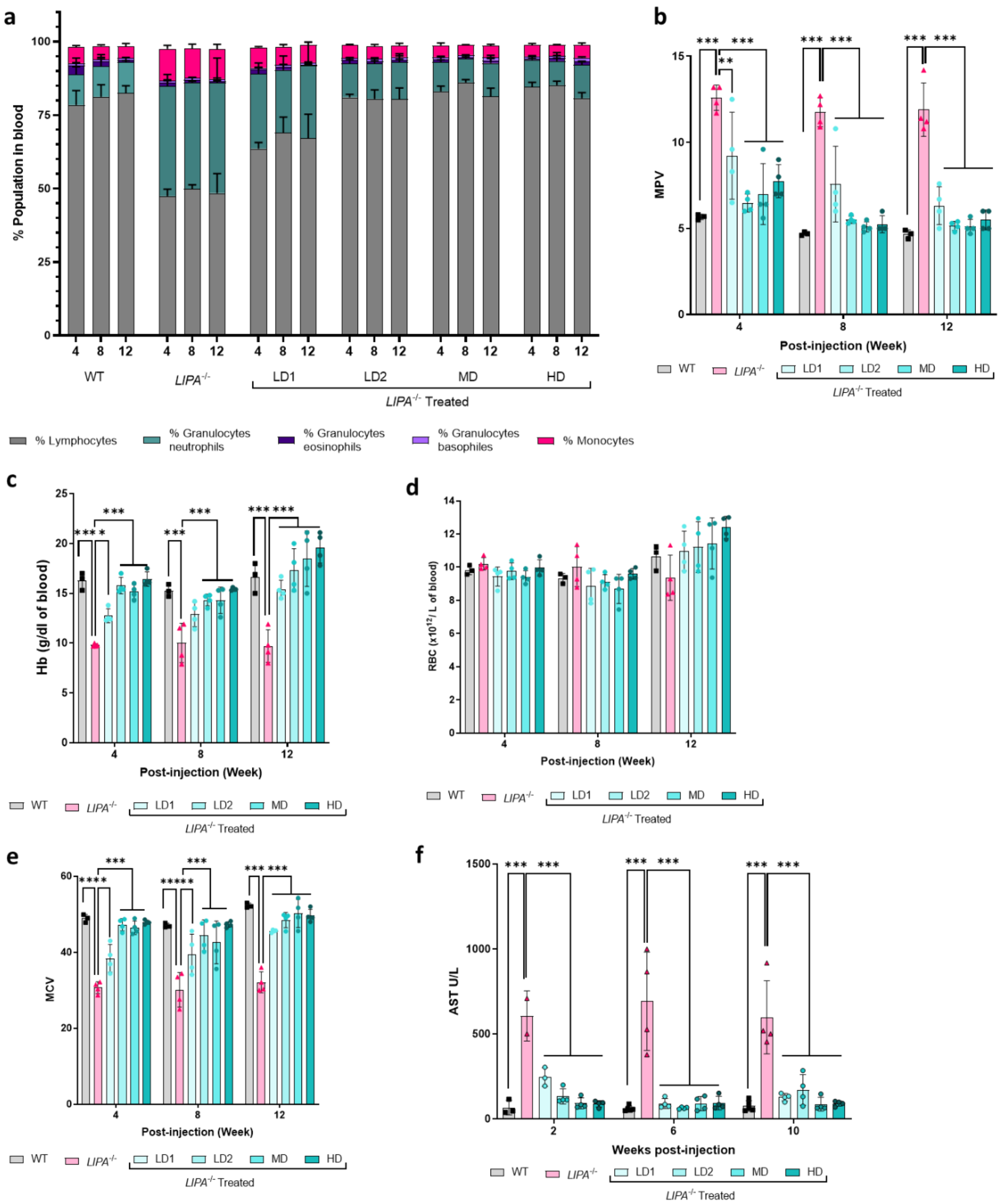

Supplementary Fig. 2. Blood analysis of *Lipa*$^{-/-}$ mice treated with 4 different rAAV8 doses. **a-b** Analysis of (**a**) blood cell populations and (**b**) mean platelet volume (MPV) at the indicated time points (n=3-4). **c-e** Analysis of red blood cells: (**c**) hemoglobin content, (**d**) count and (**e**) mean corpuscular volume (MCV) at the indicated time points (n=3-4). **f** Aspartate aminotransferase (AST) quantification (units per liter; U/L) in plasma samples at the indicate

time points (n=2-4). Bars indicate mean ± SD and statistical significance was calculated using two-way ANOVA with Tukey's test (*$p<0.033$; **$p<0.002$; *** $p<0.0001$). Exact *p* value is indicated in Supplementary Data 1.

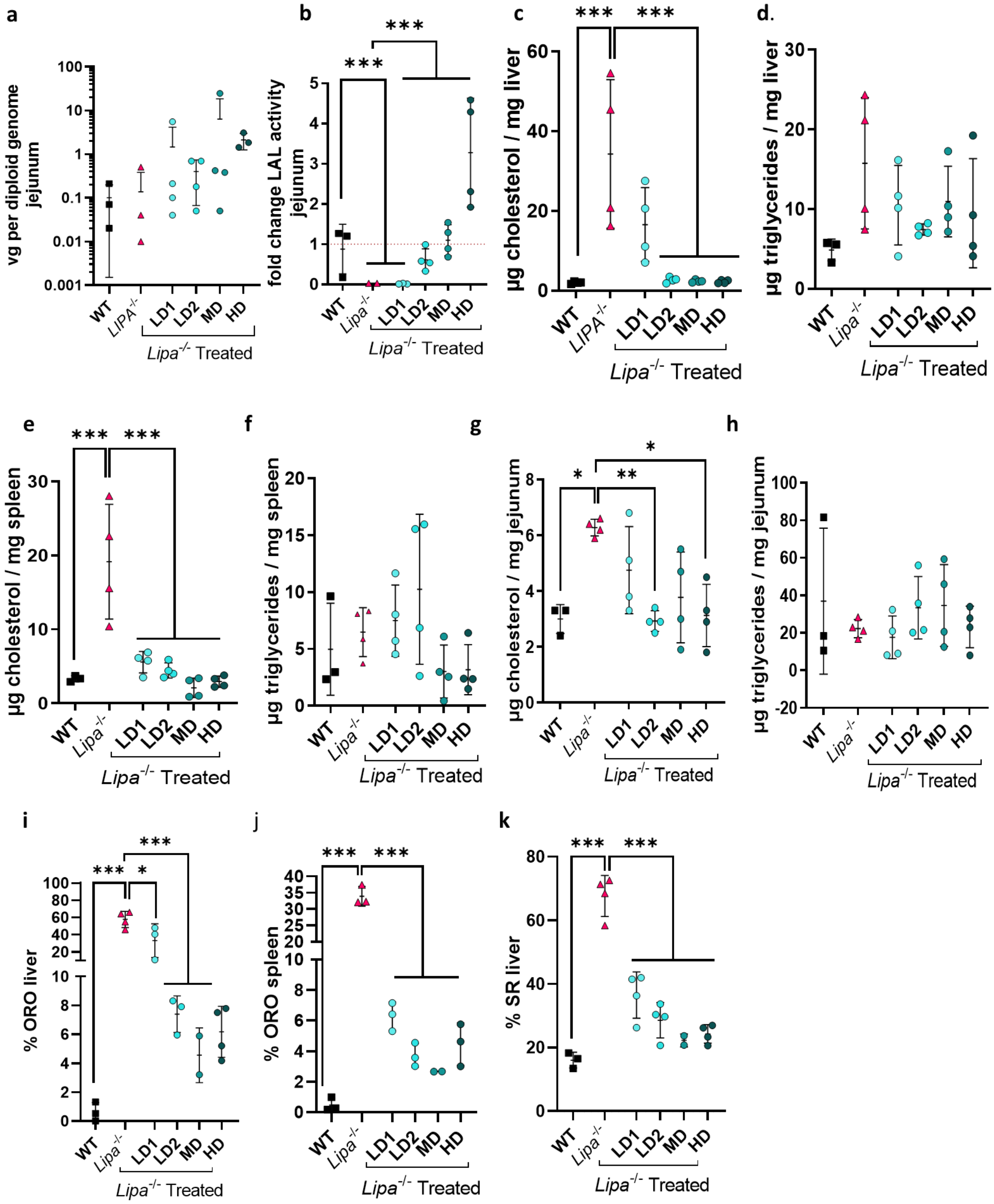

Supplementary Fig. 3. Quantification of cholesterol and triglycerides in liver, spleen, and jejunum 12-weeks post-injection. a Vector copy number of rAAV8 in jejunum (n=3-4). **b** Fold change of LAL activity over WT mice in jejunum (n=3-4). Red dotted line indicates WT

threshold. **c-d** Quantification of (**c**) cholesterol and (**d**) triglycerides per mg of liver (n=3-4). **e-f** Quantification of (**e**) cholesterol and (**f**) triglycerides per mg of spleen (n=3-4). **g-h** Quantification of (**g**) cholesterol and (**h**) triglycerides per mg of jejunum in function of the doses (n=3-4). **i-j** % of ORO-stained sections of (**i**) liver and (**j**) spleen (n=2-5). **k** % of SR-stained sections of liver (n=3-4). Bars indicate mean ± SD and statistical significance was calculated using two-way ANOVA with Tukey's test (*p<0.033; **p<0.002; *** p<0.0001). Exact *p* value is indicated in Supplementary Data 1.

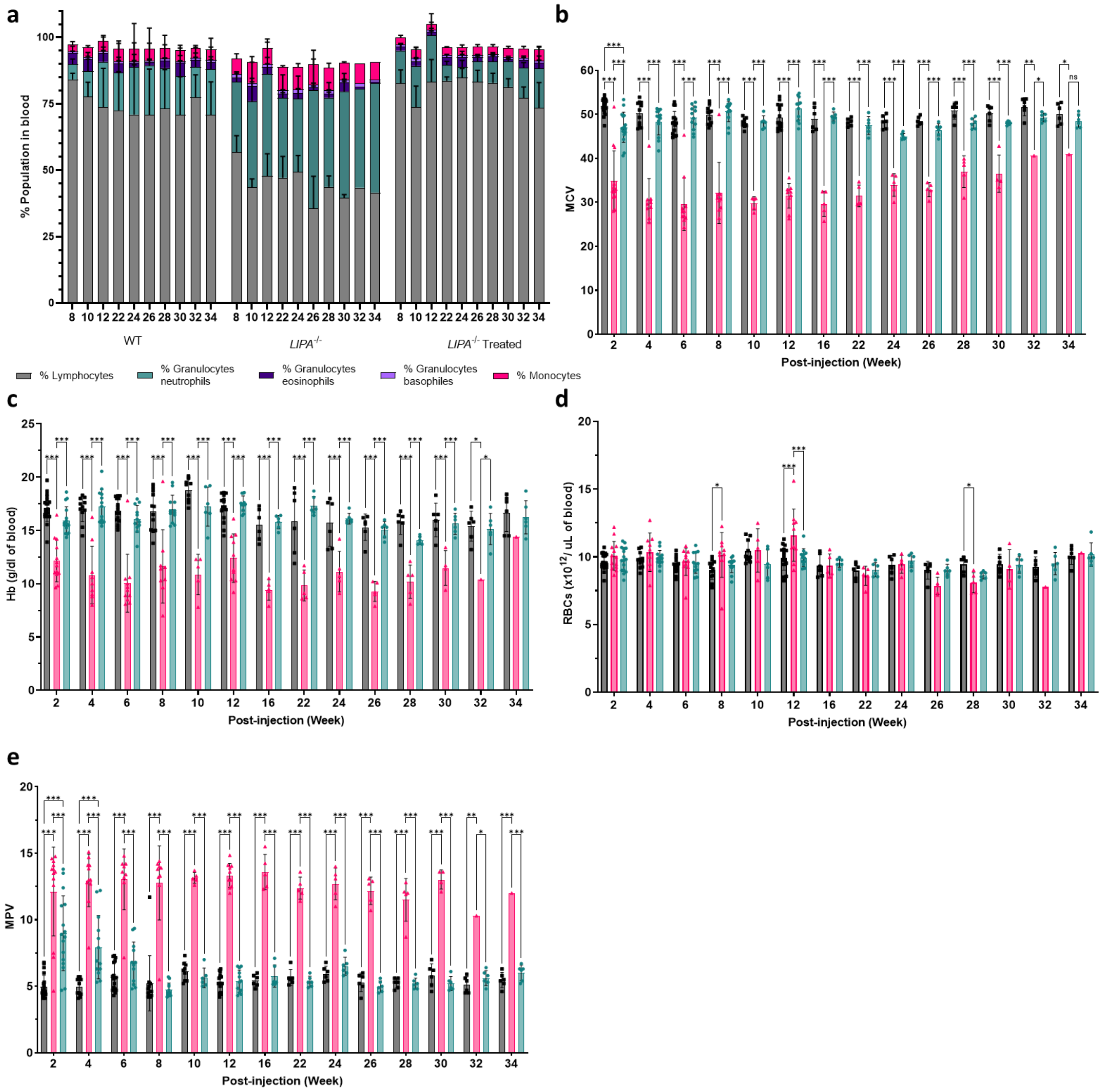

Supplementary Fig. 4. Long-term blood analysis of rAAV8.hAAT.LIPA treated *Lipa*$^{-/-}$ mice. a Blood cells composition measured at the indicated time-points (weeks). **b** Analysis of mean platelet volume (MCV) in WT, *Lipa*$^{-/-}$ and rAAV8-treated *Lipa*$^{-/-}$ mice measured at the indicated time-points (weeks). **c-e** Analysis of red blood cells: (**c**) hemoglobin (Hb) content, (**d**) RBC count and (**e**) mean corpuscular volume (MCV), measured at the indicated time-points. Bars indicate mean ± SD (n=1-20) and statistical significance was calculated using two-way

ANOVA with Tukey’s test (*$p<0.033$; **$p<0.002$; *** $p<0.0001$). Exact *p* value is indicated in Supplementary Data 1.

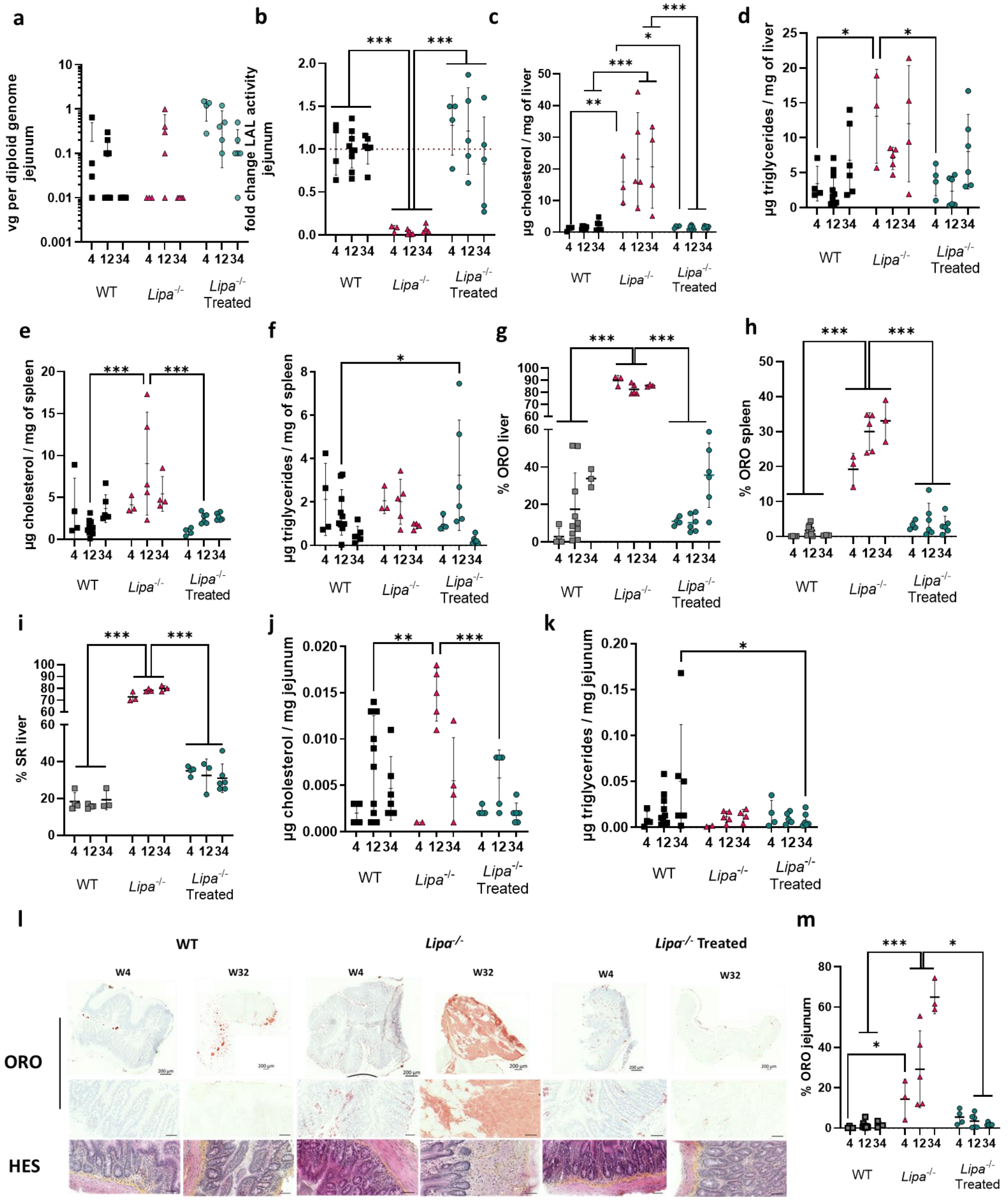

Supplementary Fig. 5. Quantification of cholesterol and triglycerides per mg of liver, spleen, and jejunum overtime. a rAAV copy number in jejunum at the indicated time-points (weeks) (n=3-9). **b** Fold change of LAL activity over WT mice at the indicated time-points (weeks) (n=3-9). Red dotted

line indicates WT threshold. **c-d** Quantification of (**c**) cholesterol and (**d**) triglycerides per mg of liver at the indicated time-points (weeks) (n=3-6). **e-f** Quantification of (**e**) cholesterol and (**f**) triglycerides per mg of spleen at the indicated time-points (weeks) (n=3-6). **g-h** % of ORO-stained sections of (**g**) liver and (**h**) spleen (n=3-9). **i** % of SR-stained sections of liver (n=2-4). **j-k** Quantification of (**j**) cholesterol and (**k**) triglycerides per mg of jejunum at the indicated time-points (weeks) (n=3-10). **l** Histological sections of jejunum at 4 and 34-weeks post-injection stained with ORO and HES (scale bar: 50µm). **m** % of ORO-stained tissues in jejunum (n=3-10). Bars indicate mean ± SD and statistical significance was calculated using two-way ANOVA with Tukey's test (*p<0.033; **p<0.002; *** p<0.0001). Exact *p* value is indicated in Supplementary Data 1.

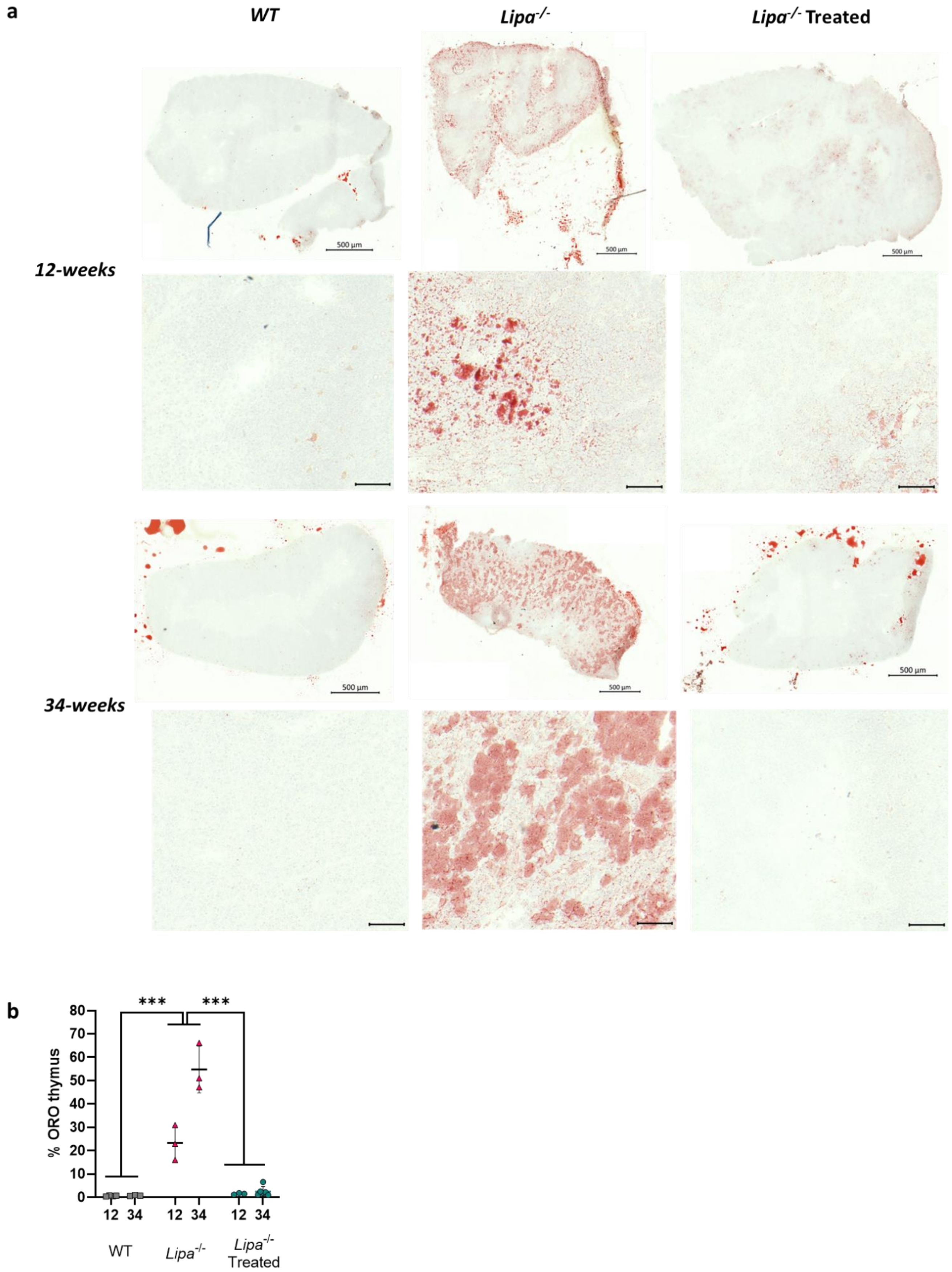
a
WT
Lipa-/-
Lipa-/- Treated
12-weeks
34-weeks
500 µm
b
***
***
% ORO thymus
0
10
20
30
40
50
60
70
80
12 34
12 34
12 34
WT
Lipa-/-
Lipa-/- Treated

Supplementary Fig. 6. Lipid deposits in thymus. a Histological section of thymus stained with ORO at 12- and 34-weeks post-injection (scale bar: 50μm). **b** % of ORO-stained sections in the thymus. Bars indicate mean ± SD (n=3-6) and statistical significance was calculated using one-way ANOVA with Tukey's test (*$p<0.033$; **$p<0.002$; *** $p<0.0001$). Exact *p* value is indicated in Supplementary Data 1.

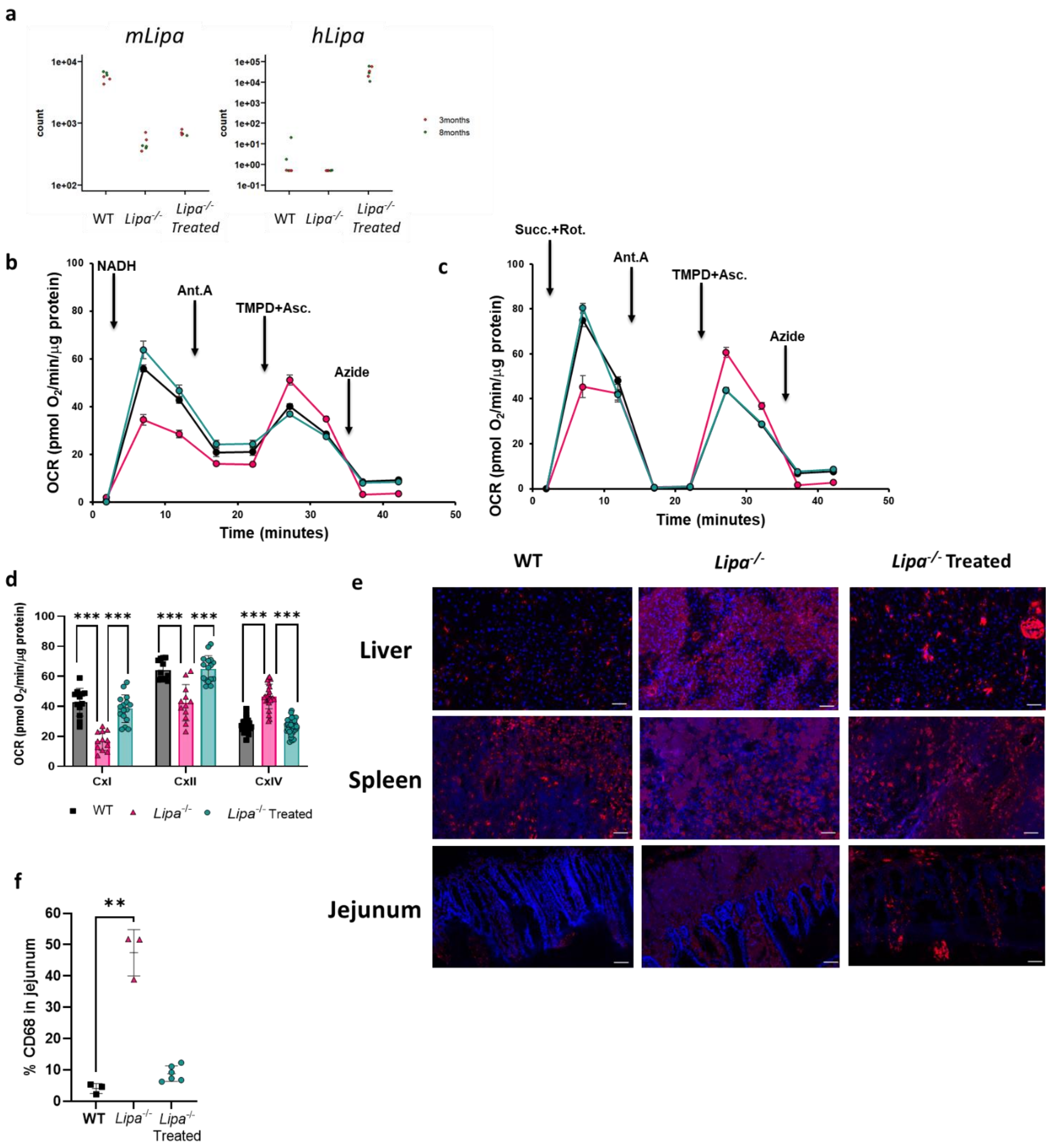

Supplementary Fig. 7. *LIPA* expressed upon rAAV8 administration corrects mitochondrial impairment in liver and immune infiltration. a Read count of murine (m) and human (h) LIPA mRNA at 34-weeks post-injection. **b-c** Representative traces of the oxygen consumption rates (OCRs) in liver of WT, *Lipa*$^{-/-}$ and rAAV8-treated *Lipa*$^{-/-}$ at 12-weeks post-treatment. Where indicated, the following compounds were injected into the assay micro-

chambers: NADH, Rotenone+Antimycin A (Rot+Ant A), Succinate+Rotenone (Succ+Rot), Antimycin A (AntA), Asc/TMPD (ascorbate+ TMPD), Azide. **d** Metabolic parameters inferred from the OCR assays and corrected for residual activity in the presence of the respiratory chain inhibitors for mitochondria isolated from mouse at 12- weeks post injection. Bars indicate mean ± SD (n = 3-4 with 3 or 4 biological replicates for each group) and statistical significance was calculated using two-way ANOVA with Bonferroni post-hoc analysis (*p<0.033; **p<0.002; *** p<0.0002). **e** Histological sections of liver (top panel), spleen (middle panel) and jejunum (bottom panel) at 34-week post-injection labelled with DAPI (blue) and murine CD68 (red) (scale: 50 µm). **f** % of mCD68-stained sections in the jejunum. Bars indicate mean ± SD (n=3-6) and statistical significance was calculated using one-way ANOVA with Kruskal-Wallis Test (*p<0.033; **p<0.002; *** p<0.0002). Exact *p* value is indicated in Supplementary Data 1.
