# Supplementary material for: Rescue of lysosomal acid lipase deficiency in mice by rAAV8 liver gene transfer
Source: Commun Med (Lond). 2025 Apr 11;5:110. doi: 10.1038/s43856-025-00816-8 (PMC11992068; doi:10.1038/s43856-025-00816-8)
Supplement: Supplementary file 2 — Description of Additional Supplementary File [file 43856_2025_816_MOESM2_ESM.pdf]

Description of additional supplementary file

File name: Supplementary Data 1
Description: source data behind graph
