# Supplementary material for: Rescue of lysosomal acid lipase deficiency in mice by rAAV8 liver gene transfer
Source: Commun Med (Lond). 2025 Apr 11;5:110. doi: 10.1038/s43856-025-00816-8 (PMC11992068; doi:10.1038/s43856-025-00816-8)
Supplement: Supplementary file 4 — reporting-summary [file 43856_2025_816_MOESM4_ESM.pdf]

nature portfolio

Corresponding author(s): Mario Amendola
mamendola@genethon.fr

Last updated by author(s): Dec 17, 2024

Reporting Summary

Nature Portfolio wishes to improve the reproducibility of the work that we publish. This form provides structure for consistency and transparency in reporting. For further information on Nature Portfolio policies, see our Editorial Policies and the Editorial Policy Checklist.

Statistics

For all statistical analyses, confirm that the following items are present in the figure legend, table legend, main text, or Methods section.

n/a	Confirmed	
☐	☒	The exact sample size (*n*) for each experimental group/condition, given as a discrete number and unit of measurement
☐	☒	A statement on whether measurements were taken from distinct samples or whether the same sample was measured repeatedly
☐	☒	The statistical test(s) used AND whether they are one- or two-sided<br>*Only common tests should be described solely by name; describe more complex techniques in the Methods section.*
☐	☒	A description of all covariates tested
☐	☒	A description of any assumptions or corrections, such as tests of normality and adjustment for multiple comparisons
☐	☒	A full description of the statistical parameters including central tendency (e.g. means) or other basic estimates (e.g. regression coefficient) AND variation (e.g. standard deviation) or associated estimates of uncertainty (e.g. confidence intervals)
☐	☒	For null hypothesis testing, the test statistic (e.g. *F*, *t*, *r*) with confidence intervals, effect sizes, degrees of freedom and *P* value noted<br>*Give P values as exact values whenever suitable.*
☒	☐	For Bayesian analysis, information on the choice of priors and Markov chain Monte Carlo settings
☒	☐	For hierarchical and complex designs, identification of the appropriate level for tests and full reporting of outcomes
☒	☐	Estimates of effect sizes (e.g. Cohen's *d*, Pearson's *r*), indicating how they were calculated

Our web collection on statistics for biologists contains articles on many of the points above.

Software and code

Policy information about availability of computer code

Data collection	Illumina, Light Cycler480, SPARK TECAN Reader, Odyssey imager, EnSpire software, Melet Schloessing blood device, FUJI DRI-CHEM NX500 - Sysmex, Axioscan Z1 slide scanner, the KingFisher™ Flex Purification System, XF96 extracellular flux analyser
Data analysis	R software, GraphPad Prism version 9.00, Microsoft Excel, Magellan Software, Zen Lite software, QuPath 0.4.3

For manuscripts utilizing custom algorithms or software that are central to the research but not yet described in published literature, software must be made available to editors and reviewers. We strongly encourage code deposition in a community repository (e.g. GitHub). See the Nature Portfolio guidelines for submitting code & software for further information.

Data

Policy information about availability of data

All manuscripts must include a data availability statement. This statement should provide the following information, where applicable:

- Accession codes, unique identifiers, or web links for publicly available datasets
- A description of any restrictions on data availability
- For clinical datasets or third party data, please ensure that the statement adheres to our policy

The authors declare that data supporting the findings of this study are available within the paper and its supplementary information files or from the corresponding author on reasonable request.
The RNA-seq data, both raw and processed ones are available on the GEO data set : GSE252742.

Human research participants

Policy information about studies involving human research participants and Sex and Gender in Research.

Reporting on sex and gender	*Use the terms sex (biological attribute) and gender (shaped by social and cultural circumstances) carefully in order to avoid confusing both terms. Indicate if findings apply to only one sex or gender; describe whether sex and gender were considered in study design whether sex and/or gender was determined based on self-reporting or assigned and methods used. Provide in the source data disaggregated sex and gender data where this information has been collected, and consent has been obtained for sharing of individual-level data; provide overall numbers in this Reporting Summary. Please state if this information has not been collected. Report sex- and gender-based analyses where performed, justify reasons for lack of sex- and gender-based analysis.*
Population characteristics	*Describe the covariate-relevant population characteristics of the human research participants (e.g. age, genotypic information, past and current diagnosis and treatment categories). If you filled out the behavioural & social sciences study design questions and have nothing to add here, write "See above."*
Recruitment	*Describe how participants were recruited. Outline any potential self-selection bias or other biases that may be present and how these are likely to impact results.*
Ethics oversight	*Identify the organization(s) that approved the study protocol.*

Note that full information on the approval of the study protocol must also be provided in the manuscript.

Field-specific reporting

Please select the one below that is the best fit for your research. If you are not sure, read the appropriate sections before making your selection.

☒ Life sciences ☐ Behavioural & social sciences ☐ Ecological, evolutionary & environmental sciences

For a reference copy of the document with all sections, see nature.com/documents/nr-reporting-summary-flat.pdf

Life sciences study design

All studies must disclose on these points even when the disclosure is negative.

Sample size	We included 3 to 4 mice in the study to ensure statistically significant results.
Data exclusions	We excluded mice that did not have been properly injected.
Replication	Experimental and biological replicates were performed for every experiment. Every measurement was performed in duplicate (technical replicates).
Randomization	Mice were randomly assigned to different treatment groups.
Blinding	Microscopy images were taken by blinded observers. Image quantification was performed by publicly available macros and softwares.

Reporting for specific materials, systems and methods

We require information from authors about some types of materials, experimental systems and methods used in many studies. Here, indicate whether each material, system or method listed is relevant to your study. If you are not sure if a list item applies to your research, read the appropriate section before selecting a response.

Materials & experimental systems

n/a	Involved in the study	
☐	☒	Antibodies
☒	☐	Eukaryotic cell lines
☒	☐	Palaeontology and archaeology
☐	☒	Animals and other organisms
☒	☐	Clinical data
☒	☐	Dual use research of concern

Methods

n/a	Involved in the study	
☒	☐	ChIP-seq
☒	☐	Flow cytometry
☒	☐	MRI-based neuroimaging

Antibodies

Antibodies used	Anti-lysosomal acid lipase antibody (ref : ab154356); Anti- α-actinin antibody (ref : sc-17829); anti-CD68 antibody (ref : MCA1957GA).
Validation	For Anti-lysosomal acid lipase antibody : Rabbit Polyclonal Lysosomal acid lipase/LAL antibody. Suitable for WB and reacts with Mouse, Human samples. Cited in 3 publications. α-actinin Antibody (H-2) is a mouse monoclonal IgG1 κ α-actinin antibody, cited in 183 publications, provided at 200 µg/ml. alpha-actinin Antibody (H-2) is recommended for detection of α-actinin isoforms of mouse, rat and human origin by WB, IP, IF, IHC(P) and ELISA. Cd68 is a rat monoclonal purified IgG clone FA-11 that recognized murine homolog of human CD68 and can be used in flow cytometry and immunofluorescence.

Animals and other research organisms

Policy information about studies involving animals; ARRIVE guidelines recommended for reporting animal research, and Sex and Gender in Research

Laboratory animals	Heterozygous Lipatm1a(EUCOMM)Hmgu/Biat mice were crossed with heterozygous mice expressing the Cre recombinase under the control of the CMV promotor to delete exon 4 of the mouse Lipa gene (11) (Fig. S.1.A). Homozygous mice were then backcrossed on the C57/BL6N background for >8 generations.
Wild animals	Wild animals were obtained from heterozygous breeding of Lipatm1a(EUCOMM)Hmgu/Biat mice.
Reporting on sex	To minimize the influence of sex on rAAV transduction (Davidoff et al., 2003) and immune reactions (Piechnik et al., 2022), male Lipa-/- mice were chosen for the study.
Field-collected samples	Mice were maintained in a specific pathogen-free (SPF) environment with a regular light-dark (12 h/12 h) cycle and ad libitum access to food (standard chow) and water.
Ethics oversight	This study was approved by the ethical committee CEEA-51 and conducted according to French and European legislation on animal experimentation (APAFIS #33620-2021101416506495).

Note that full information on the approval of the study protocol must also be provided in the manuscript.
